# Epithelial phenotype restoring drugs suppress macular degeneration phenotypes in an iPSC model

**DOI:** 10.1038/s41467-021-27488-x

**Published:** 2021-12-15

**Authors:** Ruchi Sharma, Aman George, Malika Nimmagadda, Davide Ortolan, Barbosa-Sabanero Karla, Zoya Qureshy, Devika Bose, Roba Dejene, Genqing Liang, Qin Wan, Justin Chang, Balendu Shekhar Jha, Omar Memon, Kiyoharu Joshua Miyagishima, Aaron Rising, Madhu Lal, Eric Hanson, Rebecca King, Mercedes Maria Campos, Marc Ferrer, Juan Amaral, David McGaughey, Kapil Bharti

**Affiliations:** 1 Ocular and Stem Cell Translational Research Section, National Eye Institute, National Institutes of Health, Bethesda, MD 20892 USA; 2 Pediatric, Developmental, & Genetic Ophthalmology Section, National Eye Institute, National Institutes of Health, Bethesda, MD 20892 USA; 3 Research Governance Council, FDA, White Oak, MD USA; 4 Intramural Research Program, NIAMS, National Institutes of Health, Bethesda, MD 20892 USA; 5 Division of Preclinical Innovation, NCATS, National Institutes of Health, Rockville, MD 20850 USA; 6 Histology Core, National Eye Institute, National Institutes of Health, Bethesda, MD 20892 USA; 7 Ophthalmic Genetics and Visual Functions Branch, National Eye Institute, National Institutes of Health, Bethesda, MD 20892 USA

**Keywords:** Drug discovery, Molecular biology, Stem cells, Diseases, Induced pluripotent stem cells

## Abstract

Age-related Macular Degeneration (AMD), a blinding eye disease, is characterized by pathological protein- and lipid-rich drusen deposits underneath the retinal pigment epithelium (RPE) and atrophy of the RPE monolayer in advanced disease stages - leading to photoreceptor cell death and vision loss. Currently, there are no drugs that stop drusen formation or RPE atrophy in AMD. Here we provide an iPSC-RPE AMD model that recapitulates drusen and RPE atrophy. Drusen deposition is dependent on AMD-risk-allele CFH(H/H) and anaphylatoxin triggered alternate complement signaling via the activation of NF-κB and downregulation of autophagy pathways. Through high-throughput screening we identify two drugs, L-745,870, a dopamine receptor antagonist, and aminocaproic acid, a protease inhibitor that reduce drusen deposits and restore RPE epithelial phenotype in anaphylatoxin challenged iPSC-RPE with or without the CFH(H/H) genotype. This comprehensive iPSC-RPE model replicates key AMD phenotypes, provides molecular insight into the role of CFH(H/H) risk-allele in AMD, and discovers two candidate drugs to treat AMD.

## Abstract (web summary)

Age-related macular degeneration is characterized by lipid-rich drusen deposits underneath the retinal pigment epithelium (RPE). Here the authors report an in vitro iPSC-RPE model for AMD that recapitulates drusen and RPE atrophy, and identify two drugs that reduce drusen deposits and restore RPE epithelial phenotype.

## Introduction

AMD is one of the leading causes of vision loss in adults over the age of 50^1^. It is a polygenic disease with aging and cigarette smoking as key risk factors that trigger risk alleles to manifest with age^1^. Clinically, AMD presents in two forms—wet AMD or choroidal neovascularization (CNV) and dry AMD. Dry AMD is characterized by the presence of protein and lipid-rich sub-RPE deposits called drusen. The advanced stage of dry AMD, geographic atrophy (GA), leads to vision loss and often blindness, is characterized by irreversible degeneration of the photoreceptors and retinal pigment epithelium (RPE)^2,3^. RPE is a monolayer of polarized hexagonal cells that maintain photoreceptor health by performing several functions, including nutrient and fluid transport between the photoreceptors and the choroidal blood supply and phagocytosis of worn-out photoreceptor outer segments (POS)^3^. Photoreceptor cell death in GA is initiated by functional atrophy of the RPE and the choriocapillaris, and accelerated by the eventual loss of the RPE^3,4^. Although the exact cause of RPE atrophy and cell death is not known, degenerative changes within the Bruch’s membrane and choriocapillaris leading to reduced nutrient supply is thought to damage both RPE and photoreceptor cell health^5^. There is currently no treatment available for dry AMD because the mechanism of drusen formation and RPE atrophy are not well understood.

Genome-wide association studies (GWAS) have linked several genetic variants to AMD pathogenesis^4^. Several of the variants are in genes regulating the alternate complement pathway (e.g., complement factor H (CFH), complement factor I (CFI), complement C3, complement factor B (CFB)), one of three complement pathways that leads to downstream complement effector functions via the activation of CFB mediated C3 convertase; CFH and CFI are known to inhibit this activation^6^. Activation of C3 convertase and its downstream effector C5 convertase leads to the formation of anaphylatoxins—C3a and C5a^6^. Discoveries from GWAS are substantiated by recent biomarker studies that uncovered increased expression of C3a and C5a in the eyes of AMD patients^6–9^. Furthermore, increased expression of anaphylatoxins is seen in the AMD retina and stressed RPE cells independent of their AMD-risk genotype, suggesting a locally activated alternate complement involvement in AMD^8,9^. However, how C3a and C5a proteins induce AMD pathology in the RPE is not well understood. Intracellular activity of C3a and C5a is mediated by G-protein-coupled receptors, C3aR1 and C5aR1^6^. In immune cells, the activation of these receptors activates AKT and ERK1/2 kinases, which triggers NF-κB activation leading to increased expression of inflammatory cytokines^6^. Canonical NF-κB activation from an inflammatory stimulus also leads to the inhibition of autophagy^10^, resulting in the accumulation of misfolded proteins, a potential source of sub-RPE drusen deposits^11^.

Recent use of animal models has provided critical insight into the role of complement in RPE physiology and degeneration^12–14^. Several in vitro models of AMD pathobiology have also been proposed using the primary human, mouse, pig RPE, and AMD iPSC-RPE (iRPE)^15–18^. Some of these studies were able to recapitulate sub-RPE drusen deposits. Others have linked the formation of these deposits to monogenic forms of retinal degeneration or the components of human serum, specifically, anaphylatoxin C3a^17^. However, a thorough analysis of RPE phenotype and changes in intracellular pathways induced by C3a or C5a complement proteins, leading to RPE atrophy, was not provided. Furthermore, no drug has been proposed to ameliorate the effect of complement proteins on RPE or rescue RPE atrophy seen in AMD patients.

In this work, we replicate AMD cellular phenotypes, including sub-RPE APOE and lipid deposits, and atrophy in fully mature iRPE monolayer that is derived from with CFH(H/H), CFH(Y/Y), and CFH(Y/H) variants. Complement competent human serum (CC-HS)—a source for anaphylatoxins (activated complement) is used, as a surrogate for age-induced increase in alternate complement pathway seen in AMD eyes^8,9^, to recapitulate AMD cellular phenotypes in iRPE cells. iRPE with the CFH(H/H) high-risk allele for AMD show higher baseline APOE and lipid deposition as compared to the low-risk variant CFH(Y/Y). In both CFH(Y/Y) and CFH(H/H)-iRPE, the AMD disease phenotypes are mediated by C3a–C3aR1 and C5a–C5aR1 signaling-induced NF-κB activation, autophagy downregulation, and dysregulated intracellular calcium homeostasis. A high-throughput screen using a commercially available library of pharmaceutically active compounds (LOPAC) leads to the discovery of two drugs that consistently ameliorate anaphylatoxin-induced signaling alterations in both CFH(Y/Y) and CFH(H/H)-iRPE cells and suppress AMD cellular phenotypes in both CFH(Y/Y) and CFH(H/H)-iRPE—making these drugs as likely candidates for pharmaceutical interventions of dry AMD.

## Results

### CC-HS induces AMD phenotype in iRPE cells

Published GWAS studies have demonstrated that two risk alleles have the highest associations with AMD—rs10490924, T/T in locus ARMS2/HTRA1 and rs1061170, C/C (leading to a homozygous substitution of amino acid tyrosine (Y) to histidine (H) at location 402) in gene CFH^19,20^. Here, we focused on the CFH risk allele, CFH(H/H) that increases the likelihood of dry AMD by 4.4-fold as compared to its Y/Y counterpart, whereas the heterozygous allele CFH(Y/H) provides an intermediate risk to AMD^19–21^. To develop an in vitro AMD model that works with all three categories of CFH alleles, we tested four iPSCs lines with high-risk allele CFH(H/H), three iPSC lines with the low-risk allele CFH(Y/Y), and three iPSC lines heterozygous for the allele CFH(Y/H)^21^ (Supplementary Table 1 and Table 1). To reduce the risk profile of ARMS2/HTRA1 risk allele for our analysis, we used iPSCs that were, in most cases, heterozygous for rs10490924, G/T^21^.

**Table 1:** iRPE genotyping for risk alleles for CFH, C3, and ARMS2 genes.

	CFH (Y402H) rs1061170 (T/T—nonrisk, C/C—risk)	C3 (R102G) rs2230199 (C/C—nonrisk, G/G—risk)	ARMS2 (A69S) rs10490924 (G/G—nonrisk, T/T—risk)
iRPE with CFH(Y/Y) allele			
iRPE1	T/T	C/G	G/T
iRPE2	T/T	C/G	G/G
iRPE3	T/T	C/G	G/G
iRPE with CFH(H/H) allele			
iRPE4	C/C	G/G	G/T
iRPE5	C/C	G/G	T/T
iRPE6	C/C	G/G	G/T
iRPE7	C/C	C/G	G/T
iRPE with CFH(Y/H) allele			
iRPE8	C/T	G/G	G/T
iRPE9	C/T	G/G	G/T
iRPE10	C/T	G/G	G/T

**Fig. 1:**
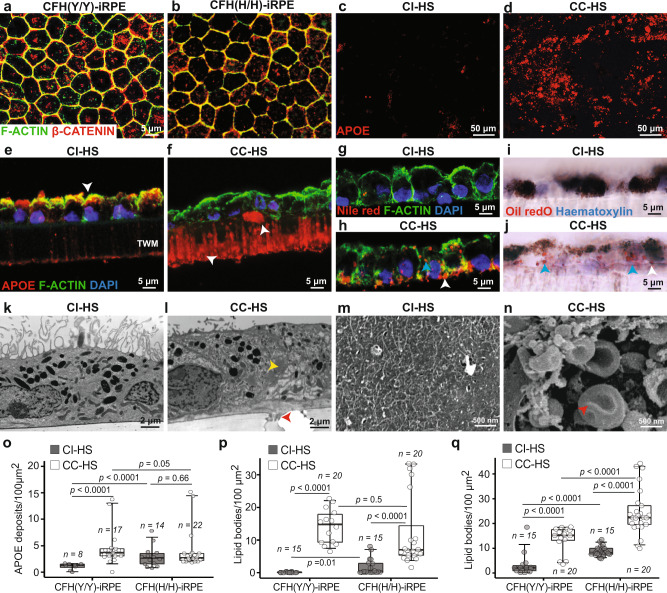
Complement competent human serum induces AMD cellular phenotypes in mature CFH(Y/Y)-iRPE and CFH(H/H)-iRPE. **a**, **b** Co-localization of F-ACTIN (green) and β-CATENIN (red) at cell borders of mature iRPE cells in CFH(Y/Y)-iRPE1 (**a**) and CFH(H/H)-iRPE6 (**b**). (*N* = 4 biologically independent replicates, iRPE1, 2, 5, 7). **c**, **d** APOE-positive deposits in CFH(H/H)-iRPE4 treated for 48 h with complement incompetent human serum (CI-HS) or complement competent human serum (CC-HS) (*N* = 7 biologically independent replicates, iRPE1, 2, 3, 4, 5, 6, 7). **e**–**j** Cross-section images of CFH(Y/Y)-iRPE1 monolayer on transwell membrane (TWM) treated with CI-HS (**e**, **g**, **i**) or CC-HS (**f**, **h**, **j**) show changed APOE localization from apical to sub-RPE zone and transwell membrane pores (arrowhead in **e**, **f**); intracellular (blue arrowhead) and sub-RPE (white arrowhead) lipid deposits stained with Nile red (**h**) and Oil Red O (**j**) (*N* = 5 biologically independent replicates, iRPE1, 4, 5, 6, 7). **k**, **l** Transmission electron micrographs of CFH(Y/Y)-iRPE2 show intracellular lipid droplets (yellow arrowhead) and sub-RPE deposits (red arrowhead) in CC-HS treated (**l**) cells (*N* = 5 biologically independent replicates, iRPE2, 4, 5, 8, 9). **m**, **n** Scanning electron micrographs of CFH(Y/Y)-iRPE2 sub-RPE surface following CI-HS or CC-HS treatment (red arrowheads show basal deposits in CC-HS treated samples) (*N* = 3 biologically independent replicates, iRPE2, 5, 8). **o**–**q** Quantification of sub-RPE APOE (**o**), BODIPY (**p**), Nile red deposits (**q**) in CFH(Y/Y)-iRPE and CFH(H/H)-iRPE under basal (CI-HS) and CC-HS treatment conditions (*N* = 7 biologically independent replicates iRPE1, 2, 3, 4, 5, 6, 7). *P* values were determined by multiple pairwise comparisons using two-tailed *t* test with 95% confidence interval and Bonferroni correction. The horizontal lines in the boxplots indicate the median, the boxes indicate the first and third quartiles, and the whiskers indicate 5th and 95th percentile.

iPSCs were differentiated into mature and polarized RPE cells using our published protocols (see “Methods” for details)^22,23^. Maturity of both CFH(Y/Y) and CFH(H/H) iRPE was confirmed by the nuclear to cell membrane translocation of β-CATENIN (Fig. 1a, b), suggesting the formation of tight junctions between neighboring cells^22^ iRPE polarization was confirmed by apically located EZRIN, a marker of RPE apical processes (Supplementary Fig. 1a–d). Both CFH(Y/Y)-iRPE and CFH(H/H)-iRPE showed a similar pattern of β-CATENIN and EZRIN localization in mature iRPE (Fig. 1a, b and Supplementary Fig. 1a–d), suggesting comparable maturation and polarization of iRPE with the two CFH genotypes. To test our hypothesis of a critical role of anaphylatoxins in AMD pathogenesis, we treated mature iRPE monolayer derived from CFH(Y/Y) and CFH(H/H) donors with complement competent human serum (CC-HS) with activated anaphylatoxins or complement incompetent human serum (CI-HS) with an inhibited activity of anaphylatoxins^15^. CC-HS treatment led to a dramatic increase in APOE-positive sub-RPE deposits both in CFH(Y/Y)-iRPE and CFH(H/H)-iRPE (Fig. 1c, d). Cross-section views of iRPE monolayer revealed a significant change in APOE localization from predominantly apical in CI-HS treated samples to a strong sub-cellular signal that was seen both above and inside the membrane (white arrowheads, Fig. 1e, f), similar to the previous observations^15,16^. Consistent with drusen deposits seen in AMD eyes and in RPE cultures^15,24^, APOE-positive deposits co-stained with an anti-membrane attack complex (C5b-9) antibody (Supplementary Fig. 1e). iRPE monolayer cross and en face sections stained with Nile red and Oil Red O revealed increased intracellular and sub-RPE accumulation of lipids in CC-HS-treated iRPE as compared to CI-HS treated cells (blue arrowhead points to intracellular signal and white to sub-RPE signal—Fig. 1g–j and Supplementary Fig. 1f–i—white circles show intracellular signal)^16,25^. The presence of intracellular lipid deposits in CC-HS-treated iRPE cells was further confirmed using transmission electron microscopy (TEM) (yellow arrowhead, Fig. 1l, compared to 1k). Sub-RPE deposits seen in TEM (red arrowhead, Fig. 1l) were further characterized by hematoxylin and eosin (H&E) staining (red arrowheads, Supplementary Fig. 1j–m) and high-magnification TEM images (red arrowheads, Supplementary Fig. 1n–q). TEM clearly showed that deposits were located under the RPE cell basal membrane (blue arrowheads, Supplementary Fig. 1n–q). Scanning electron microscopy (SEM) further confirmed the presence of sub-RPE, often dome-shaped, deposits in CC-HS-treated samples (red arrowheads, Fig. 1m, n and Supplementary Fig. 1r–u). Together, these findings support our hypothesis that CC-HS treatment induces hallmark disease phenotypes of AMD in iRPE monolayer derived from both CFH(Y/Y) and CFH(H/H) donors.

We hypothesized that AMD-associated CFH risk allele (Y402H)^19,20^ in iRPE cells locally controls the activation of C3 into anaphylatoxin C3a and predisposes CFH(H/H)-iRPE cells toward diseases. Under basal (CI-HS treated) conditions, CFH(H/H)-iRPE formed 2× higher APOE deposits as compared to CFH(Y/Y)-iRPE (*P* < 0.001; Fig. 1o) and these numbers in CFH(H/H)-iRPE did not change significantly under CC-HS conditions (*P* = ns; Fig. 1o). In comparison, CFH(Y/Y)-iRPE cells formed 2× higher APOE under CC-HS as compared to CI-HS-treated conditions (*P* < 0.001; Fig. 1o). Similar to the APOE data, lipid accumulation—as quantified by BODIPY and Nile red stains—was between 2 and 10× higher in CFH(H/H)-iRPE under basal conditions as compared to CFH(Y/Y)-iRPE (*P* < 0.05 for Fig. 1p and *P* < 0.001 for Fig. 1q). Both BODIPY (8–14×, *P* < 0.001) and Nile red (3–4×, *P* < 0.001) signals increased in both CFH(H/H)-iRPE and CFH(Y/Y)-iRPE under CC-HS conditions (Fig. 1p, q). Taken together, these results suggest that, as compared to CFH(Y/Y)-iRPE, CFH(H/H)-iRPE show higher disease susceptibility at the baseline. CC-HS increases lipid deposits in both genotypes albeit to varying degrees allowing us to discern specific effects of AMD genotype on disease phenotypes.

### CC-HS induces atrophy in CFH(Y/Y) and CFH (H/H)-iRPE cells

**Fig. 2:**
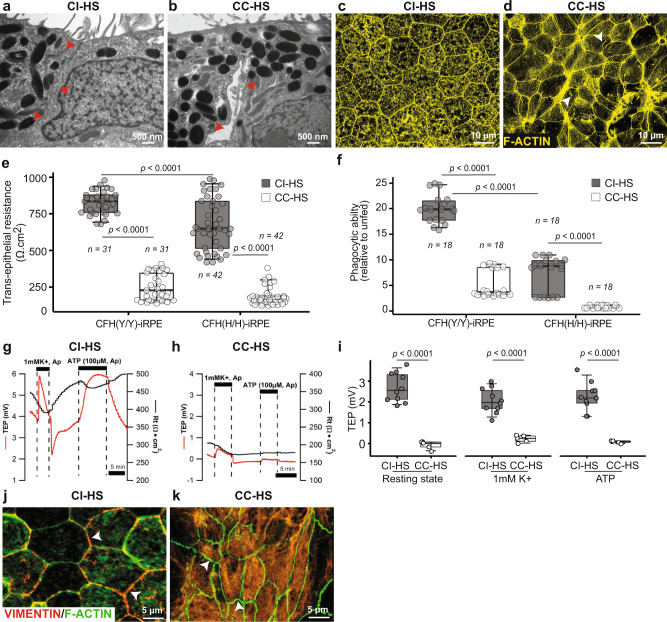
Complement competent human serum induces RPE atrophy in CFH(Y/Y)-iRPE and CFH(H/H)-iRPE. **a**, **b** Transmission electron micrographs show disrupted tight junctions in CC-HS treated CFH(Y/Y)-iRPE2 as compared to CI-HS treated CFH(Y/Y)-iRPE2 (red arrowheads) (*N* = 4 biologically independent replicates, iRPE2, 4, 8, 9). **c**, **d** CC-HS treatment-induced stress fibers (arrowheads in **d**) and resulted in the loss of hexagonal morphology (F-ACTIN, yellow) in cells, compared to CI-HS treatment of CFH(H/H)-iRPE5 (*N* = 3 biologically independent replicates, iRPE5, 8, 9). **e**, **f** Transepithelial resistance (TER or Rt) (**e**) and phagocytic ability (**f**) of CFH(Y/Y)-iRPE and CFH(H/H)-iRPE under basal (CI-HS) and CC-HS treatment conditions (*N* = 7 biologically independent replicates iRPE1, 2, 3, 4, 5, 6, 7) for TER and phagocytosis. *P* values were determined by pairwise comparisons using two-tailed *t* test with 95% confidence interval and Bonferroni correction. The horizontal lines in the boxplots indicate the median, the boxes indicate the first and third quartiles, and the whiskers indicate 5th and 95th percentile. **g**–**i** Resting stage and physiological stimuli of apical 1 mM K + and 100 mM ATP induced transepithelial potential (TEP) of CI-HS and CC-HS-treated iRPE monolayers. Representative traces are shown in (**g**, **h**), quantification is shown in (**i**) (*N* = 10 independent experiments for CI-HS and *N* = 5 independent experiments for CC-HS, iRPE8). *P* values were determined by pairwise comparisons using two-tailed *t* test with 95% confidence interval and Bonferroni correction. The horizontal lines in the boxplots indicate the median, the boxes indicate the first and third quartiles, and the whiskers indicate 5th and 95th percentile. **j**, **k** Immunostaining for VIMENTIN (red) in CI-HS-treated CFH(Y/Y)-iRPE2 (**j**) and CC-HS-treated CFH(Y/Y)-iRPE1 (**k**). Arrowheads point to membrane staining in control cells that is missing in CC-HS treated iRPE (*N* = 6 biologically independent replicates, iRPE1, 3, 5, 6, 7, 8).

The junctional integrity of RPE is critical in maintaining the epithelial phenotype^22^. Loss of epithelial phenotype and RPE atrophy are seen in AMD eyes^26,27^. We asked if CC-HS treatment induces atrophy in this iRPE model via the loss of RPE junctional integrity. TEM of CC-HS-treated cells revealed disintegrated junctional complexes between neighboring RPE cells derived from CFH(Y/Y) and CFH(H/H)-iPSCs (arrowhead in Fig. 2a, b). To further investigate the integrity of the tight junctions between adjacent RPE cells in CC-HS-treated samples, we stained the cells for CLDN19—a tight junction protein^24^. CLDN19 was missing from cell borders and localized intracellularly in CC-HS-treated CFH(Y/Y) and CFH(H/H)-iRPE samples (arrowheads in Supplementary Fig. 2a, b). F-ACTIN staining displayed intracellular stress fibers in CC-HS-treated iRPE cells that failed to retain their characteristic hexagonal morphology reminiscent of transition from AMD phenotype 2 to phenotype 3 when cells start losing their characteristic epithelial phenotype^27^ (arrowheads in Fig. 2d). We asked whether these structural changes in RPE cell morphology translated into functional differences in their ability to maintain tight junctions—as measured by TER of the monolayer. The baseline TER of CFH(H/H)-iRPE was 25% lower as compared to the TER of CFH(Y/Y)-iRPE (*P* < 0.001; Fig. 2e). Upon CC-HS stimulation CFH(H/H)-iRPE saw a drop of 85% in TER, whereas TER of CFH(Y/Y)-iRPE dropped by 70% (*P* < 0.001; Fig. 2e). Under baseline conditions, we noticed a 50% lower ability of CFH(H/H)-iRPE cells to phagocytose photoreceptor outer segments (POS) conditions as compared to CFH(Y/Y)-iRPE cells (*P* < 0.001; Fig. 2f). Upon CC-HS stimulation, both CFH(Y/Y)-iRPE and CFH(H/H)-iRPE further lost their ability to phagocytose POS by five- to sixfold (*P* < 0.001; Fig. 2f). These results suggest that CFH(Y/Y)-iRPE and CFH(H/H)-iRPE have baseline differences in their functional capacity and it deteriorates further in both genotypes under the complement challenge similar to what is seen in AMD eyes.

Consistent with an eroded epithelial phenotype, CC-HS-treated CFH(Y/Y) and CFH(H/H)-RPE lost their polarized status; as demonstrated by a reduced steady-state transepithelial potential^28^ (TEP—measures the difference in apical and basal membrane potential and reflects enhanced functional polarization and maturation of the RPE monolayer; TEP 4 mV vs 0.25 mV, *P* < 0.001), lower hyperpolarization response to a physiological stimulus of reducing apical K + concentration from 5 to 1 mM (2 mV vs 0.5 mV, *P* < 0.001), and a negligible depolarization response to an apical ATP stimulus known to activate calcium signaling, ion, and fluid absorption in the RPE^28,29^ (*P* < 0.001; Fig. 2g–i).

To further dissect the mechanism of RPE atrophy, we stained iRPE cells for VIMENTIN—a dedifferentiation marker present in AMD RPE cells^30,31^. VIMENTIN expression was predominantly on the cell membrane in CI-HS and non-treated CFH(Y/Y) and CFH(H/H) iRPE cells but was missing from the cell membrane and did not form an organized cytoskeleton network in elongated CC-HS treated CFH(Y/Y) and CFH(H/H) iRPE cells (arrowheads, Fig. 2j, k and Supplementary Fig. 2c, d), similar to RPE dedifferentiation seen at the borders of GA lesion in cadaver AMD eyes (Supplementary Fig. 2e–h). Overall, our results showed that CC-HS treatment induces the most notable features seen in AMD RPE—the formation of APOE and lipid-containing sub-cellular deposits, and RPE atrophy through cellular dedifferentiation—a phenotype associated with advanced disease stages.

### CC-HS triggers C3a/C5aR1 signaling in iRPE cells

The anaphylatoxin arm of the complement pathway acts via C3a–C3aR1 and C5a–C5aR1 signaling^6^. We checked if the CC-HS treatment led to changes in expression of CFH and the two anaphylatoxins in CFH(Y/Y)-iRPE and CFH(H/H)-iRPE. ELISA showed that as compared to CI-HS treatment, CC-HS treatment led to 24–30-fold higher CFH secretion on the basal, but not the apical, side of CFH(Y/Y) and CFH(H/H)-iRPE [30-fold for CFH(Y/Y) and 24-fold for CFH(H/H), *P* < 0.001; Supplementary Fig. 3a). CC-HS also led to several fold higher apical (C3a:7-fold for CFH(Y/Y), *P* < 0.001 and 5-fold for CFH(H/H), *P* < 0.05; C5a: threefold for CFH(Y/Y), *P* < 0.001 and 1.7-fold for CFH(H/H), *P* = ns) and basal (C3a: 1.4-fold for CFH(Y/Y), *P* < 0.05 and 5.6-fold for CFH(H/H), *P* < 0.05; C5a: 1.2-fold for CFH(Y/Y), *P* < 0.05 and threefold for CFH(H/H), *P* = ns) levels of C3a and C5a (Supplementary Fig. 3b, c). Baseline or CC-HS induced differences between CFH(Y/Y) and CFH(H/H)-iRPE were not significant. Overall, these results confirmed that CC-HS treatment causes RPE cells to activate the alternate complement pathway in a cell-autonomous fashion that likely contributes to RPE pathology.

**Fig. 3:**
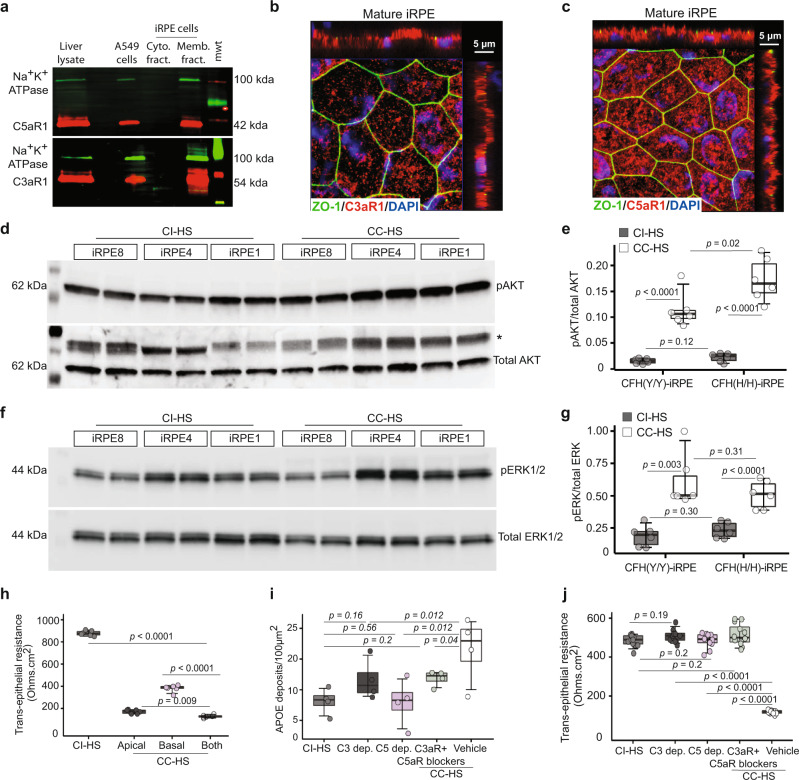
AMD cellular phenotypes in CFH(Y/Y)-iRPE and CFH(H/H)-iRPE are induced by C3a–C3aR1 and C5a–C5aR1 signaling. **a** Western blot of membrane and cytoplasmic fractions confirms the presence of complement receptor 3a (C3aR1) and complement receptor 5a (C5aR1) in iRPE membranes. Liver and A549 cell line cell lysates served as positive controls. Na^+^/K^+^ ATPase was used as a loading control (*N* = 3 biologically independent replicates, iRPE1, 5, 8). **b**, **c** Predominant apical localization of C3aR1 (red) (**b**) and C5aR1 (red) (**c**) in CFH(H/H)-iRPE5 cells. ZO-1 (green) marks apical tight junctions (*N* = 4 biologically independent replicates, iRPE1, 5, 8, 9). **d**–**g** Western blots (**d**, **f**) and their quantification (**e**, **g**) demonstrate 7-d-fold upregulation of pAKT (**d**, **e**) and two- to threefold upregulation of pERK (**f**, **g**) in CC-HS treated iRPE cells, compared to CI-HS-treated iRPE cells (*N* = 6 biologically independent replicates and *n* = 2 technical replicates, iRPE1, 2, 3, 4, 5, 7 for **e**, **g**). *P* values were determined by multiple pairwise comparisons using two-tailed *t* test with 95% confidence interval and Bonferroni correction. The horizontal lines in the boxplots indicate the median, the boxes indicate the first and third quartiles, and the whiskers indicate 5th and 95th percentile. **h** TER measurements after 48 h of CI-HS or CC-HS treatment on apical only, basal only, and combined apical/basal sides of iRPE. (*N* = 3 independent experiments and *n* = 2 technical replicates; iRPE6, 8). *P* values were determined by pairwise comparisons using two-tailed *t* test with 95% confidence interval and Bonferroni correction. The horizontal lines in the boxplots indicate the median, the boxes indicate the first and third quartiles, and the whiskers indicate 5th and 95th percentile. **i**, **j** APOE-positive sub-RPE deposits (**i**) and TER measurement (**j**) across iRPE monolayers treated with CI-HS, C3, or C5-depleted sera, CC-HS and receptor blockers for C3aR1 (PMX53 10 μM) and C5aR1 (compstatin 10 μM), CC-HS + vehicle (vehicle). *N* = 4 independent experiments for (**i**). *N* = 3 biologically independent replicates and *N* = 4 independent experiments, iRPE1, 5, 6, for (**j**); additional iRPE samples in Supplementary Fig. 3t, u). *P* values were determined by pairwise comparisons using two-tailed *t* test with 95% confidence interval and Bonferroni correction. The horizontal lines in the boxplots indicate the median, the boxes indicate the first and third quartiles, and the whiskers indicate 5th and 95th percentile.

To decipher the mechanism of C3a and C5a signaling mediated AMD phenotype induction in iRPE cells, we checked the expression and localization of receptors, C3aR1 and C5aR1. RNAseq confirmed the expression of both receptors in iRPE cells with ~30× higher expression of C5aR1 as compared to C3aR1 (Supplementary Fig. 3d). Western blot showed the presence of C3aR1 and C5aR1 receptors in the membrane fraction of iRPE cells (Fig. 3a). Immunostaining of mature iRPE cells and RPE from cadaver human eyes confirmed the localization of C3aR1 and C5aR1 both on the apical and the basal sides of RPE cells, with stronger labeling in iRPE on the apical side (Fig. 3b, c and Supplementary Fig. 3e–n). To verify that CC-HS activates C3aR1 and C5aR1 in iRPE cells, we analyzed phosphorylation of AKT and ERK1/2, the two key kinases downstream of C3aR1 and C5aR1 receptors^32^. Western blot for CC-HS treated samples from CFH(Y/Y)-iRPE and CFH(H/H)-iRPE showed 7-8x increase in levels of pAKT (*P* < 0.001) and 2–3× increase in levels of p-ERK1/2 (*P* < 0.001) as compared to CI-HS treated cells (Fig. 3d–g). Furthermore, consistent with the dominant apical localization of C3aR1 and C5aR1 in iRPE, apical only treatment of CC-HS was sufficient to cause the 4–5× TER drop (*P* < 0.001) caused by the combined apical/basal treatment. In contrast, the basal only treatment of CC-HS resulted in a twofold TER drop (*P* < 0.01; Fig. 3h). Lower efficacy of CC-HS on the basal iRPE side is also consistent with over 200× increase in the basal secretion of CFH upon CC-HS treatment, whereas no increase in apical-side CFH secretion was noted (Supplementary Fig. 3a). To further dissect the role of C3aR1 and C5aR1 signaling in inducing AMD phenotypes in iRPE cells, we employed depleted sera and receptor blocker strategy. Treatment of CFH(Y/H), CFH(Y/Y), and CFH(H/H)-iRPE cells with sera depleted in C3 (*P* < 0.05 for CFH(Y/H); *P* = ns for CFH(Y/Y); *P* < 0.001 for CFH(H/H)) or C5 (*P* < 0.05 for CFH(Y/H); *P* = ns for CFH(Y/Y); *P* < 0.001 for CFH(H/H)) proteins, or the concurrent use of blockers for C3aR1 (compstatin, 10 μM)^33^ and C5aR1 (PMX053, 10 μM)^6^ in CC-HS serum (*P* < 0.05) resulted in two- to fivefold lower sub-RPE APOE deposits as compared to CC-HS plus vehicle treatment (Fig. 3i and Supplementary Fig. 3o–t). Depletion of complement factor D, an upstream regulator of C3a and C5a formation, also led to lower sub-RPE APOE deposits as compared to complete CC-HS medium^32^ (Supplementary Fig. 3s). Similarly, as compared to CI-HS treated samples, no changes in TER were observed in CFH(Y/H), CFH(Y/Y), and CFH(H/H)-iRPE monolayer treated with sera depleted in C3 (*P* < 0.001) or C5 (*P* < 0.001) or in iRPE cotreated with CC-HS and blockers for C3aR1 and C5aR1 receptors (compstatin, 10 μM and PMX053, 10 μM respectively; *P* < 0.001; Fig. 3j; Supplementary Fig. 3u). In summary, our data indicate that stimulation of C3aR1 and C5aR1 complement receptors on iRPE monolayers is required for triggering AMD phenotype in both CFH(Y/Y)-iRPE and CFH(H/H)-iRPE.

### CC-HS activates NF-κB and suppresses autophagy pathways

**Fig. 4:**
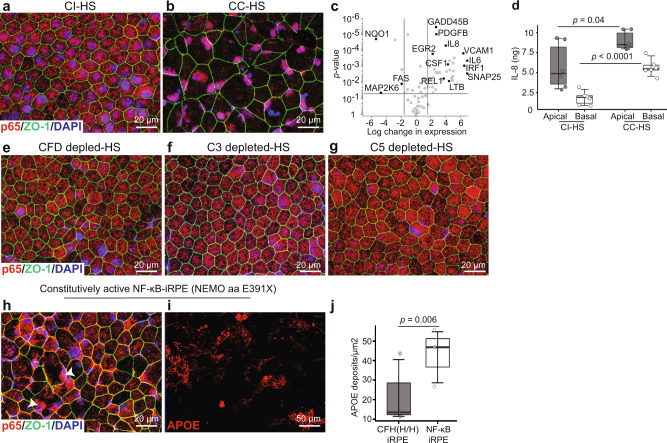
Activation of NF-κB pathway downstream of C5aR1 and C3aR1 signaling leads to sub-RPE APOE deposits in CFH(Y/Y)-iRPE and CFH(H/H)-iRPE. **a**, **b** Nuclear translocation of p65 (red) in CC-HS treated CFH(Y/Y)-iRPE2 cells shows NF-κB activation. ZO-1 (green) (*N* = 7 biologically independent experiments, iRPE1, 2, 3, 4, 5, 6, 8). **c** qRT-PCR confirms increased expression of NF-κB target genes in CC-HS treated iRPE (*N* = 4 independent experiments, iRPE1, 8). Genes with more than 4× expression changes and *P* < 0.05 are deemed significant. Exact *P* values are plotted in the figure, *P* values were determined by pairwise comparisons using two-tailed *t* test with 95% confidence interval and Bonferroni correction. **d** ELISA shows CC-HS-treated iRPE have twofold higher apical and basal secretion of IL-8, an NF-κB target cytokine (*N* = 3 independent experiments and N = 2 biological replicates, iRPE1, 9). *P* values were determined by pairwise comparisons using two-tailed *t* test with 95% confidence interval and Bonferroni correction. The horizontal lines in the boxplots indicate the median, the boxes indicate the first and third quartiles, and the whiskers indicate 5th and 95th percentile. **e**–**g** CFH(Y/Y)-iRPE2 cells treated with sera depleted in complement factor D (CFD), C3, or C5 do not show nuclear translocation of p65 (red), ZO-1 (green) (*N* = 7 biologically independent replicates, iRPE1, 2, 3, 5, 6, 7, 8). **h** CI-HS treated iRPE derived from a patient with E391X mutation in protein NEMO demonstrate constitutive nuclear localization of p65 (red, arrowhead) ZO-1 (green), (*N* = 3 independent experiments, NEMO-iRPE). **i**, **j** APOE (red) positive sub-RPE deposits in NEMO (E391X)-iRPE treated with CI-HS as compared to CI-HS treatment in CFH(H/H)-iRPE5 (**j**) (*N* = 3 independent experiments, NEMO-iRPE and iRPE5. *P* value was determined by pairwise comparisons using two-tailed *t* test with 95% confidence interval and Bonferroni correction. The horizontal lines in the boxplots indicate the median, the boxes indicate the first and third quartiles, and the whiskers indicate 5th and 95th percentile.

To conduct an unbiased analysis of pathways affected by C3a–C3aR1 and C5a–C5aR1 signaling, we performed RNAseq analysis of CI-HS and CC-HS-treated iRPE derived from CFH(Y/Y), CFH(H/H), and CFH(Y/H)-iPSCs. Principal component analysis of the data revealed a globally different gene expression pattern induced by CC-HS treatment and showed similar changes across iRPE derived from the three genotypes (Supplementary Fig. 4a— heatmap shows sample distance). Consistent with the effect of anaphylatoxins C3a, C5a in immune cells^6,32,34^, autophagy (*P* < 0.001) and TNF/NF-κB (*P* < 0.001) were the pathways most affected by CC-HS treatment in iRPE with both CFH(Y/Y) and (H/H) alleles (Supplementary Fig. 4b). This led us to hypothesize that C3aR1-C3a and C5aR1-C5a signaling in iRPE cells is a pivotal regulator of these two pathways. Indeed, CC-HS treatment caused the p65 (RELA) subunit of NF-κB to translocate to the nucleus in both CFH(Y/Y) and CFH(H/H)-iRPE cells (Fig. 4a, b and Supplementary Fig. 4d–o). Furthermore, CC-HS treatment increased the expression of NF-κB target genes in both CFH(Y/Y) and CFH(H/H)-iRPE^35^—as confirmed by (1) RNAseq (Supplementary Fig. 4c, *P* < 0.001); (2) qRT-PCR for IL-6, IL-8, GADD45B, EGR2, NF-κB1A, REL1, NF-κB1, SNAP25 (Fig. 4c, *P* < 0.01–*P* < 0.001); (3) immunostaining for RELB and TRAF3 (Supplementary Fig. 4p–s); and (4) a twofold increased secretion of NK-κB pathway inflammatory cytokines, IL-8 (Fig. 3d, apical *P* < 0.05, basal *P* < 0.001) and IL-18 (Supplementary Fig. 4t, apical *P* < 0.05, basal *P* < 0.05).

Predominant cytoplasmic localization of p65 in CFH(Y/Y), CFH(H/H), and CFH(Y/H)-iRPE cells treated with sera depleted in CFD, C3, or C5 proteins provided further evidence that anaphylatoxins directly activate the NF-κB pathway in iRPE cells (Fig. 4e–g and Supplementary Fig. 5a–r). To determine if NF-κB activation directly led to the formation of sub-RPE APOE-positive deposits, we derived iRPE cells from a patient with an E391X truncating mutation in the NEMO gene—a negative regulator of NF-κB signaling^36^. Consistent with the literature, constitutive nuclear translocation of p65 was observed in NEMO mutant iRPE cells even under CI-HS treatment (Fig. 4h). In addition, a 5–6× increase in sub-RPE APOE-positive deposits were detected in mutant iRPE cells under CI-HS treatment conditions (*P* < 0.001; Fig. 4i, j). Overall, these results demonstrate that the anaphylatoxins-triggered AMD disease phenotypes in iRPE cells are mediated through the activation of the NF-κB pathway.

**Fig. 5:**
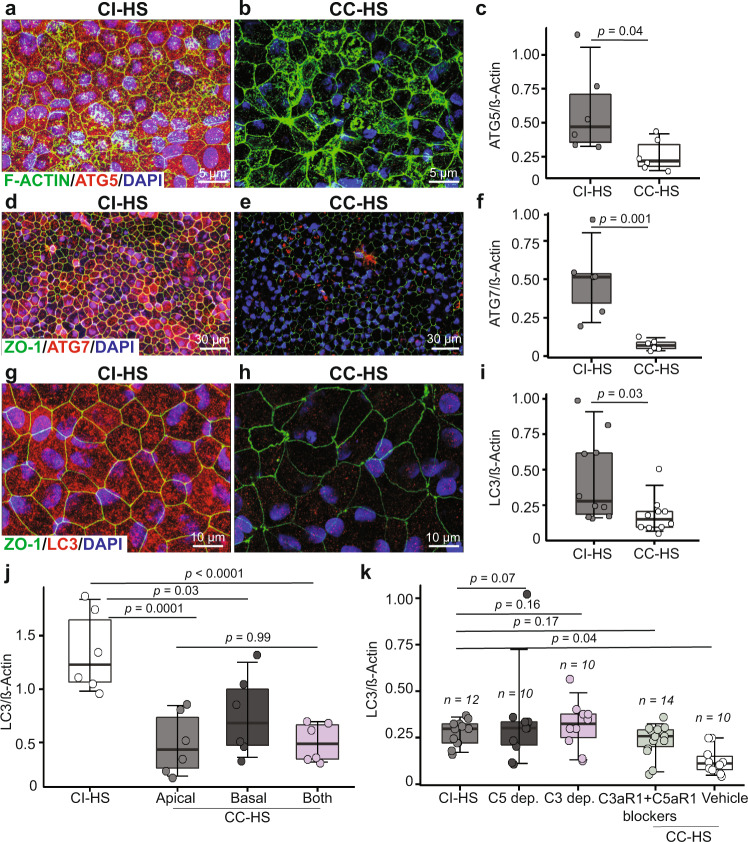
Anaphylatoxin signaling downregulates autophagy in CFH(Y/Y)-iRPE and CFH(H/H)-iRPE iRPE cells. **a**–**i** Autophagy pathway proteins, ATG5 (red, **a**, **b**), ATG7 (red, **d**, **e**) and LC3 (red, **g**, **h**), are downregulated in CC-HS-treated CFH(Y/Y)-iRPE2, compared to CI-HS treatment; as confirmed by immunostaining (**a**–**h**, F-ACTIN—green), and quantification of western blots for ATG5 (**c**), ATG7 (**f**) and LC3-II (**i**) (*N* = 3 biologically independent replicates and two technical replicates, iRPE1, 4, 8 for (**a**–**h**); *N* = 5 biologically independent replicates and two technical replicates, iRPE1, 4, 7, 8, 10 for (**i**)). *P* values were determined by pairwise comparisons using two-tailed *t* test with 95% confidence interval and Bonferroni correction. The horizontal lines in the boxplots indicate the median, the boxes indicate the first and third quartiles, and the whiskers indicate 5th and 95th percentile. **j** Western blot quantification shows similar decrease in LC3-II levels in iRPE treated with apical only and both apical and basal sides CC-HS treatment. In contrast, basal only CC-HS treatment shows lower LC3-II signal (*N* = 6 independent replicates and three experiments, iRPE1). *P* values were determined by multiple pairwise comparisons using two-tailed *t* test with 95% confidence interval and Bonferroni correction. The horizontal lines in the boxplots indicate the median, the boxes indicate the first and third quartiles, and the whiskers indicate 5th and 95th percentile. **k** Western blot quantification shows LC3-II levels are unaffected in iRPE treated with CI-HS, sera depleted in C5 or C3, or when cotreated with CC-HS and receptor blockers for C3aR1 (PMX53 10 μM) and C5aR1 (compstatin 10 μM), compared to CC-HS + vehicle (vehicle) treated samples (*N* = 5 biologically independent replicates, iRPE1, 4, 5, 8, 10; additional iRPE samples in Supplementary Fig. 6t). *P* values were determined by multiple pairwise comparisons using two-tailed *t* test with 95% confidence interval and Bonferroni correction. The horizontal lines in the boxplots indicate the median, the boxes indicate the first and third quartiles, and the whiskers indicate 5th and 95th percentile.

RNAseq analysis also revealed statistically significant (*P* < 0.001) downregulation of autophagy pathway genes in CC-HS treated CFH(Y/Y) and CFH(H/H)-iRPE (Supplementary Fig. 4b). This prompted us to investigate the role of autophagy in CC-HS-induced AMD phenotypes in iRPE. Immunostaining and western blot analyses revealed that genes integral for autophagy regulation, ATG5, ATG7, and LC3-II, were all three- to fourfold downregulated in CC-HS-treated CFH(Y/Y), CFH(Y/H), and CFH(H/H)-iRPE, compared to CI-HS-treated iRPE cells (*P* < 0.01; Fig. 5a–i and Supplementary Fig. 6a–n). These results were further corroborated by qRT-PCR, which showed 4–16-fold downregulated expression of critical autophagy pathway genes (*P* < 0.01–0.001 for ATG3, ATG12, ATG4B, ATG4D, BCL2, LAMP1, SQSTM1, MAP1LC3A, MAP1LC3B) in CC-HS-treated iRPE cells, as compared to CI-HS-treated iRPE cells (Supplementary Fig. 6o). Reduced expression of key autophagy genes in CC-HS treated cells suggested reduced autophagy flux, which was confirmed by increased accumulation of autophagosomes inside CC-HS-treated iRPE cells (Supplementary Fig. 6p, q, arrowhead and inset with higher magnification)^37^.

Consistent with the dominant apical localization of C3aR1 and C5aR1 in iRPE, treatment of CC-HS on the apical side (*P* < 0.001) only of CFH(Y/Y) and CFH(H/H)-iRPE was sufficient to induce downregulation of LC3-II, a major marker for autophagy (*P* = ns between both sides CC-HS and apical only CC-HS treatment; Fig. 5j and Supplementary Fig. 6r). Furthermore, unlike CC-HS-treated cells, CFH(Y/Y) and CFH(H/H)-iRPE cells treated with CC-HS sera depleted in C3 or C5 proteins were incapable of inducing autophagy downregulation and performed similarly to CI-HS (*P* < 0.05 CI-HS vs CC-HS; *P* = ns CI-HS vs C5 depl. or C3 dept CC-HS; Fig. 5k, and Supplementary Fig. 6s, t). Similarly, iRPE cells cotreated with CC-HS and C3aR1 + C5aR1 blockers behaved similar to CI-HS-treated samples and did not show a statistically significant reduction in LC3 levels (*P* = ns for CI-HS vs C3aR1 + C5aR1 blockers + CC-HS; Fig. 5k, and Supplementary Fig. 6s). These results confirm that the anaphylatoxin signaling C3aR1 and C5aR1 inhibits autophagy in CC-HS-treated iRPE cells.

To further understand the sequence of events leading to the formation of sub-RPE APOE deposits, we performed a temporal analysis of NF-κB activation and autophagy downregulation after the addition of CC-HS on iRPE cells. Within 6 h of CC-HS addition, nuclear translocation of p65 was evident in a few cells, and over 24 h, this translocation was observed in most of the cells (Supplementary Fig. 7a–e). Similarly, LC3-II downregulation could be seen within 6 h of CC-HS treatment peaking by 24 h (Supplementary Fig. 7f–k). In contrast, a 3.7× increase in APOE deposits was observed only after 48 h (Supplementary Fig. 7l-o), indicating that NF-κB upregulation and autophagy downregulation precede the formation of APOE deposits in CC-HS-treated iRPE cells.

### High-throughput screen discovered drugs rescue iRPE atrophy

**Fig. 6:**
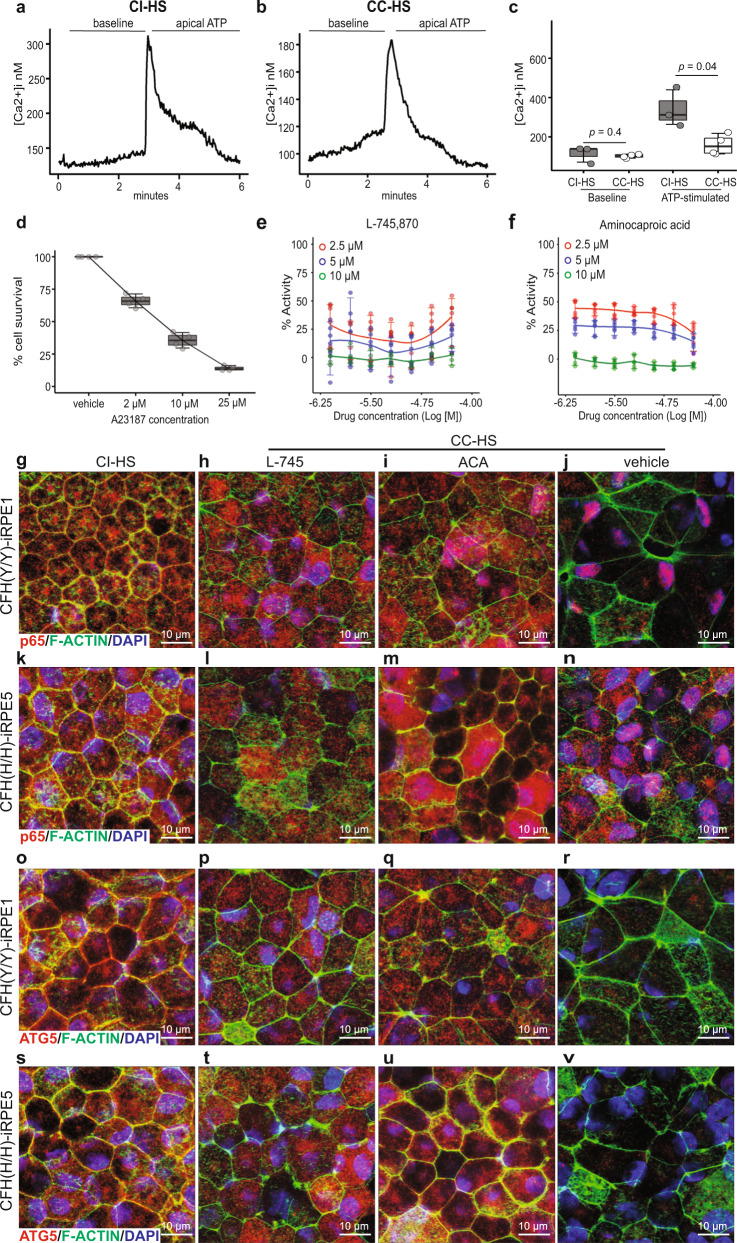
High-throughput screen identifies drugs that restore NF-κB and autophagy pathways activity in CC-HS-treated CFH(Y/Y)-iRPE and CFH(H/H)-iRPE. **a**–**c** Representative traces for intracellular Ca^2+^ ([Ca^2+^]_i_) changes in response to ATP stimuli in CI-HS- and CC-HS-treated iRPE cells (**a**, **b**; *N* = 4 independent experiments, iRPE8). **c** Boxplot shows similar baseline calcium levels and twofold lower ATP induced cytosolic calcium-level increase in CC-HS treated as compared to CI-HS treated iRPE (*N* = 4 independent experiments, iRPE8). *P* values were determined by pairwise comparisons using two-tailed *t* test with 95% confidence interval and Bonferroni correction. The horizontal lines in the boxplots indicate the median, the boxes indicate the first and third quartiles, and the whiskers indicate 5th and 95th percentile. **d** Survival curve of iRPE cells treated with calcium ionophore, A23187, performed at concentrations of 0, 2, 10, and 25 μM (*N* = 8 independent experiments, 8 technical replicates, iRPE4). **e**, **f** Seven-point dose response curves for L745 (**e**) and ACA (**f**), show reproducible cell survival across iRPE1 and 4, and three different A23187 concentrations (2.5 μM, red; 5 μM, blue; and 10 μM, green), *N* = 3 independent experiments. Error bars represent 90% confidence interval. **g**–**n** L745 and ACA suppress nuclear translocation of p65 (red) induced by CC-HS treatment on CFH(Y/Y)-iRPE and CFH(H/H)-iRPE; F-ACTIN (green), DAPI (blue). (*N* = 4 biologically independent replicates, iRPE1, 2, 5, 6). **o**–**v** L745 and ACA increase ATG5 (red) expression in CFH(Y/Y)-iRPE and CFH(H/H)-iRPE cells treated with CC-HS. F-ACTIN (green), DAPI (blue) (*N* = 4 biologically independent replicates, iRPE1, 2, 5, 6).

RNAseq revealed a defect in the intracellular calcium homeostasis pathway in CC-HS-treated cells (Supplementary Fig. 4b). This observation was supported by literature evidence of sub-RPE calcium nodules associated with AMD^38^ and by intracellular calcium-level measurements in CC-HS- or CI-HS-treated iRPE cells. The baseline calcium levels in CI-HS- and CC-HS-treated cells were similar (100–120 nM; Fig. 6a–c). But, the application of extracellular ATP, a key signaling molecule in numerous RPE cellular processes, only weakly activated the release of calcium from intracellular stores in CC-HS-treated cells resulting in a two and a half-fold lower ATP-evoked [Ca^2+^]_i_ response compared to CI-HS-treated cells (Fig. 6a–c, *P* < 0.05). Calcium is a key mediator of RPE junctional integrity and several intracellular signaling pathways, leading us to hypothesize that calcium homeostasis defects are central to AMD cellular phenotypes seen in CC-HS-treated cells. To test this hypothesis, we designed a drug screen around calcium homeostasis defect-induced iRPE cell death. A calcium ionophore A23187, similar to CC-HS treatment leads to calcium homeostasis defect and RPE cell death was used for the drug screen^39^ (Fig. 6d and Supplementary Fig. 8a). At 96 h, all three concentrations of A23187 (2.5, 10, 25 μM) led to >75% cell death, at 48 h, 2.5 μM caused 50% cell death, and 10 μM caused 70% cell death (Fig. 6d and Supplementary Fig. 8a). In total, 10 μM concentration and 48-h treatment of A23187 were selected for the drug screening because it provided a larger window for cell death rescue.

A commercially available library of pharmaceutically active compounds (LOPAC) with 1280 drugs was used for the screen at two different concentrations, 9.2 μM and 46 μM. Drug treatment, along with the stressor, 10 μM A23187, were added on iRPE cells matured in 384-well plates, and cell death was scored 48 h later by ATP release using the CellTitrGlo^TM^ assay^40^. Comparable relative mean intensity of signal across all the assay plates containing A23187 confirmed consistency and reproducibility of the screen (Supplementary Fig. 8b). Normalized cell death signal exhibited that plates 7–10, with the lower drug concentration (9.2 μM), had reduced cell death as compared to plates with the higher drug concentration (46 μM) (Supplementary Fig. 8c). At 9.2 μM 45 drugs showed improved (>40%) cell survival in A23187-treated cells (Supplementary Fig. 8d).

A detailed analysis of the drug data revealed potential artifacts in some of the 384-well plate lanes that might have contributed to false-positive signals (circled in Supplementary Fig. 8d). To distinguish false positives from the real signals and confirm drug efficacy across iRPE with both low and high-risk for AMD, we performed a follow-up screen on the 45 identified candidate drugs using CFH(Y/Y)-iRPE and CFH(H/H)-iRPE. The follow-up screen was performed at three different A23187 concentrations (2.5, 5, 10 μM) and with seven different concentrations of drugs ranging from 0.78 to 50 μM (Supplementary Fig. 8e–g). Only two drugs, L745,870 (L745) and aminocaproic acid (ACA) (Fig. 6e, f), exhibited reproducible cytoprotective activity across both CFH(Y/Y)-iRPE and CFH(H/H)-iRPE, confirming our initial observation of false-positive effects across several 384-well plate lanes (Supplementary Fig. 8d).

### Drugs reverse CC-HS-induced pathway changes in iRPE cells

Based on the seven-point dose response curve performed on CFH(Y/Y)-iRPE and CFH(H/H)-iRPE cells (Fig. 6e, f), we selected the IC_50_ dose for the two drugs (6 μM for L745 and 30 μM for ACA) to co-treat iRPE cells along with CC-HS. Immunostaining for p65 revealed reduced nuclear localization in both CFH(Y/Y) and CFH(H/H)-iRPE cotreated with CC-HS and L745 or ACA, as compared to CC-HS and DMSO (vehicle) cotreated samples (Fig. 6g–n and Supplementary Fig. 9a–h). RNAseq further confirmed that cotreatment of CFH(Y/Y) and CFH(H/H)-iRPE with CC-HS and L745 or ACA reversed gene expression changes induced by CC-HS treatment (Supplementary Fig. 10a) and resulted in up to 16-fold reduced expression of NF-κB pathway genes as compared to CC-HS and vehicle co- samples (*P* < 0.001 and Supplementary Fig. 10b). Consistently, expression of autophagy genes increased in CC-HS + drug-treated CFH(Y/Y)-iRPE and CFH(H/H)-iRPE cells as confirmed by increased immunostaining for ATG5 (Fig. 6o–v and Supplementary Fig. 9i–p) and up to tenfold higher expression of autophagy genes in RNAseq (Supplementary Fig. 10c, *P* < 0.001). Overall, these results demonstrated that the two drugs discovered in our HTS were able to reverse intracellular pathways that mediated anaphylatoxin-induced AMD phenotype in iRPE cells with both CFH(Y/Y) and CFH(H/H) genotypes. These results prompted us to further test the effect of these drugs on AMD cellular phenotypes, RPE-epithelial atrophy, and cellular functions.

### Drugs ameliorate AMD phenotypes in iRPE cells

**Fig. 7:**
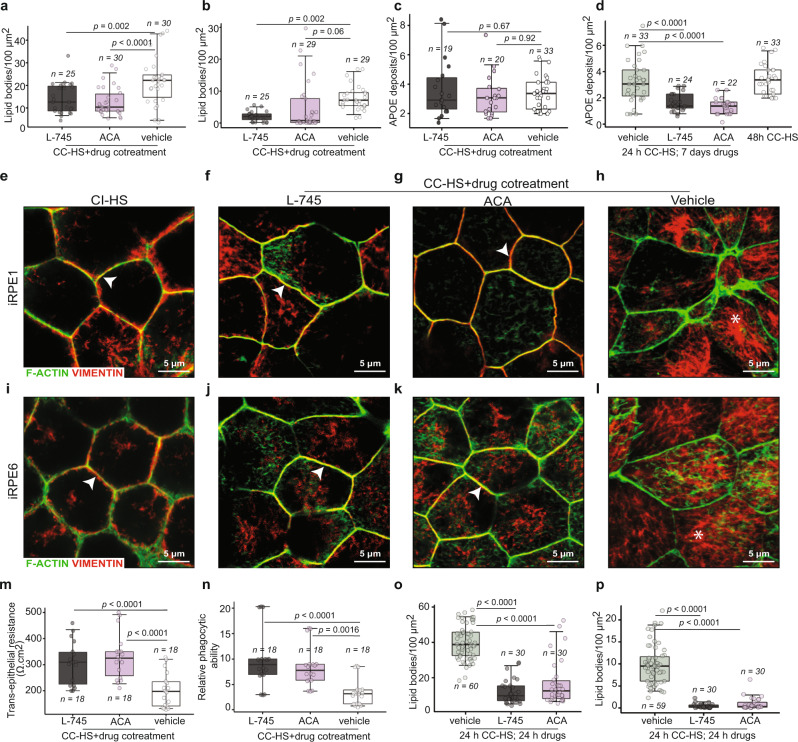
L745 and ACA restore epithelial phenotype in CC-HS-treated CFH(Y/Y)-iRPE and CFH(H/H)-iRPE. **a**–**c** Changes in levels of Nile red (**a**), BODIPY (**b**), and APOE (**c**) stained sub-RPE deposits in CFH(Y/Y), CFH(Y/H), CFH(H/H) cotreated with CC-HS plus L745 or ACA, compared to CC-HS plus vehicle cotreated cells (*N* = 6 biologically independent replicates, iRPE1, 4, 5, 6, 7, 8). *P* values were determined by multiple pairwise comparisons using two-tailed *t* test with 95% confidence interval and Bonferroni correction. The horizontal lines in the boxplots indicate the median, the boxes indicate the first and third quartiles, and the whiskers indicate 5th and 95th percentile. **d** APOE-positive sub-RPE deposits in CFH(Y/Y), CFH(Y/H), CFH(H/H) treated with vehicle, L745 or ACA for 7 days after 24 h CC-HS treatment, compared with a 48-h CC-HS + vehicle treatment (*N* = 6 biologically independent replicates, iRPE1, 4, 5, 6, 7, 8). *P* values were determined by multiple pairwise comparisons using two-tailed *t* test with 95% confidence interval and Bonferroni correction. The horizontal lines in the boxplots indicate the median, the boxes indicate the first and third quartiles, and the whiskers indicate 5th and 95th percentile. **e**–**l** Immunostaining for VIMENTIN (red) shows localization defect induced by CC-HS treatment is restored by cotreatment of CC-HS with L745 or ACA. F-ACTIN (green) stains the cytoskeleton (*N* = 5 biologically independent replicates, iRPE1, 3, 5, 6, 7). **m, n** Cotreatment of CC-HS with drugs L745 or ACA rescued CC-HS induced TER drop (**m**) and improved phagocytic ability (**n**) in CFH(Y/Y) and CFH(H/H)-iRPE (*N* = 6 biologically independent replicates, iRPE1, 2, 3, 5, 6, 7). *P* values were determined by multiple pairwise comparisons using two-tailed *t* test with 95% confidence interval and Bonferroni correction. The horizontal lines in the boxplots indicate the median, the boxes indicate the first and third quartiles, and the whiskers indicate 5th and 95th percentile. **o**, **p** Nile red (**o**) and BODIPY (**p**) positive sub-RPE lipid deposits in CFH(Y/Y) and CFH(H/H)-iRPE treated with vehicle, L745 or ACA for 24 h after a 24-h CC-HS treatment (*N* = 6 biologically independent replicates, 1, 4, 5, 6, 7, 8). *P* values were determined by multiple pairwise comparisons using two-tailed *t* test with 95% confidence interval and Bonferroni correction. The horizontal lines in the boxplots indicate the median, the boxes indicate the first and third quartiles, and the whiskers indicate 5th and 95th percentile.

The hallmark AMD cellular phenotypes seen in cadaver eyes and observed in our in vitro model are the sub-RPE lipid and protein-rich deposits, loss of RPE-epithelial phenotype, and RPE dedifferentiation that leads to atrophy of cells^41,42^. We tested if the two drugs could rescue any of these phenotypes in CC-HS-treated cells derived from CFH(Y/Y) and CFH(H/H) iPSCs. CFH(Y/Y) and CFH(H/H)-iRPE cotreated with CC-HS and L745 or ACA had 40–60% lower levels of cholesterol and triglycerides deposits as compared to CC-HS + vehicle-treated samples, as measured by Nile red staining (Fig. 7a; CC-HS + vehicle vs CC-HS + L745, *P* < 0.01; CC-HS + vehicle vs CC-HS + ACA, *P* < 0.001). Similarly, the cotreatment of CFH(Y/Y)-iRPE and CFH(H/H)-iRPE with CC-HS and L745 led to a 4× reduction in lipids, as measured by BODIPY staining (Fig. 7b; CC-HS + vehicle vs CC-HS + L745, *P* < 0.01; Supplementary Fig. 11a–c). But the levels of APOE-positive deposits did not change significantly upon a 48-h cotreatment of L745 or ACA together with CC-HS (Fig. 7c). This led us to test whether a longer-term drug treatment could reduce sub-RPE APOE-positive deposits. Indeed, a 7-day drug treatment that started 24 h after CC-HS was able reduce APOE deposits by up to twofold by both drugs (Fig. 7d; CC-HS + vehicle vs CC-HS + L745, *P* < 0.001; CC-HS + vehicle vs CC-HS + ACA, *P* < 0.001; Supplementary Fig. 11d–g).

Loss of RPE-epithelial phenotype was analyzed by VIMENTIN expression in cells. Misshapen, less compact cells with higher cytoplasmic (less membrane) expression of dedifferentiation markers like VIMENTIN are considered to have lost the epithelial phenotype and undergoing dedifferentiation^31^. Cotreatment of iRPE with CC-HS treated plus L745 or ACA reduced the cytoplasmic expression (star in Fig. 7h, l) of VIMENTIN in both CFH(Y/Y)-iRPE and CFH(H/H)-iRPE and increased its membrane expression (arrowheads in Fig. 7e–g, i–k) similar to what is seen in CI-HS-treated and non-treated iRPE (Fig. 7e, i and Supplementary Fig. 2c, d). Consistently RNAseq of CFH(Y/Y)-iRPE and CFH(H/H)-iRPE treated with CC-HS plus the vehicle or cotreated with CC-HS and the two drugs revealed four- to tenfold lower expression of dedifferentiation markers in CC-HS + drug-treated iRPE, as compared to CC-HS + vehicle-treated cells (Supplementary Fig. 11h). Combined together immunostaining and RNAseq data suggested the two drugs are able to successfully restore epithelial phenotype both in CFH(Y/Y)-iRPE and CFH(H/H)-iRPE.

Rescue of epithelial morphology prompted us to check if the two drugs rescued RPE functionality in CC-HS treated cells. TER, a measure of junctional integrity, of CFH(Y/Y) and CFH(H/H)-iRPE monolayers cotreated with drugs and CC-HS, was two- to threefold higher (*P* < 0.001) as compared to iRPE treated with CC-HS + vehicle (Fig. 7m and Supplementary Fig. 11i). Furthermore, the addition of either drug improved (up to twofold; *P* < 0.001) the ability of CFH(Y/Y) and CFH(H/H)-iRPE cells to phagocytose POS, as compared to cells treated with CC-HS and the vehicle (Fig. 7n and Supplementary Fig. 11j). To further validate the ability of these drugs as potential therapies for AMD, we tested whether treatment of these drugs after the start of the complement insult would halt disease progression. In fact, a 24-h treatment of L745 or ACA started 24-h after the start of CC-HS treatment is sufficient to cause a 3–4× reduction in lipid deposits as confirmed by Nile red staining (Fig. 7o; CC-HS + vehicle vs CC-HS + L745, *P* < 0.001; CC-HS + vehicle vs CC-HS + ACA, *P* < 0.001) and a 9× reduction in lipids, as confirmed by BODIPY staining (Fig. 7p; CC-HS + vehicle vs CC-HS + L745, *P* < 0.001; CC-HS + vehicle vs CC-HS + ACA, *P* < 0.001; Supplementary Fig. 11k–m).

In conclusion, these results confirmed that both drugs, L745 and ACA, effectively reversed AMD cellular phenotypes seen in CC-HS treated CFH(Y/Y)-iRPE and CFH(H/H)-iRPE cells. These drugs also reversed RPE atrophy and restored its functionality. Our in vitro data provide evidence to support the potential use of these drugs to delay AMD disease initiation and slow its progression on iRPE with high-risk alleles for AMD.

## Discussion

**Fig. 8:**
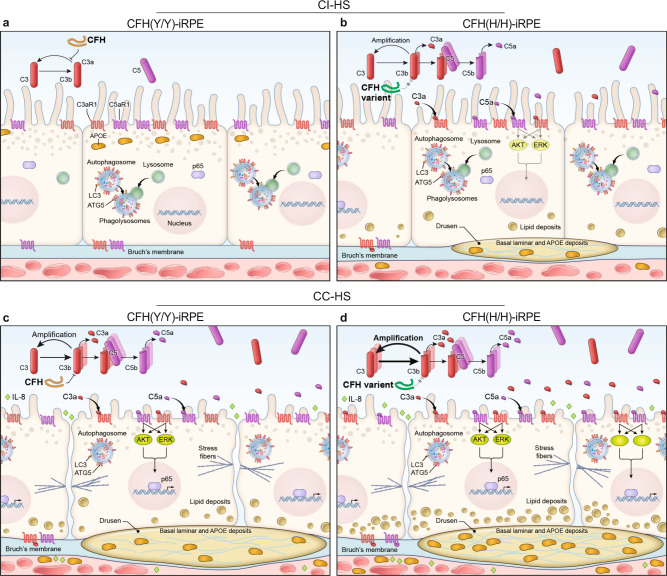
Schematic of CI-HS and CC-HS-treated CFH(Y/Y)-iRPE and CFH(H/H)-iRPE. **a**–**d** Schematic of CFH(Y/Y)-RPE (**a**, **c**) and CFH(H/H)-iRPE (**b**, **d**) following CI-HS (**a**, **b**) or CC-HS (**c**, **d**) treatments. Under basal (CI-HS) conditions, CFH(H/H)-iRPE show higher tendency for sub-RPE deposits as compared to CFH(Y/Y)-iRPE, likely because of increased alternate complement activation around RPE cells (**a**, **b**). Enhanced activation of human complement (CC-HS) further exaggerates AMD cellular phenotypes in iRPE cells; CFH(H/H)-iRPE show predisposition to disease as compared to CFH(Y/Y)-iRPE (**c**, **d**).

In a human iPSC-derived platform, our work replicates two key cellular phenotypes of AMD—sub-RPE deposits and RPE atrophy. We demonstrate that these phenotypes are associated with one of the highest risk alleles for AMD, CFH(H/H). Activated alternate complement exaggerates AMD cellular phenotypes in high-risk iRPE underscoring the crosstalk between AMD genetics and environmental factors (Fig. 8a–d)^9,43^. Our work also unveiled two clinically tested drugs that rescue AMD phenotypes in both CFH(H/H) and CFH(Y/Y) alleles, making them strong candidates with the potential to work on diverse AMD genotypes. The establishment of this complement-based iRPE model, its direct connection to the CFH risk allele and with readouts directly linked to AMD cellular phenotypes in the RPE forms a basis for testing additional anti-AMD drugs and discovering the role of other AMD-risk alleles in disease etiology.

Our working hypothesis is that there is a local source (likely retinal and RPE) of C3a and/or C5a leading to the activation of corresponding receptors C3aR1 and C5aR1 on the RPE surface. Our ELISA data show a cell-autonomous C3a and C5a source in stressed RPE cells (Supplementary Fig. 3a–c). Several previous publications support this hypothesis and suggest aged/stressed RPE and photoreceptors as potential sources of activated complement: (1) Katschke et al. recently demonstrated that POS could locally activate complement in the retina^9^. Strong immunostaining for C3 could be detected in POS of AMD eyes; (2) POS as a source of C3 and C5 leading to RPE atrophy is consistent with a previous observation of recurrence of RPE atrophy in dry-AMD patients after a full macular translocation procedure^44^; (3) Multiple reports have demonstrated that RPE cells exposed to physiological stressors can be an autonomous source of complement C3 and C3a^45–47^. Although all this work doesn’t rule out an alternative complement source coming from the systemic circulation or the choroid, likely through “leaky” endothelial cells, our data and these published studies favor a local complement source^48^.

RPE cells also express several complement inhibitors, including CD46, CD55, CFD, CFH, and CFI (Supplementary Fig. 12), which can suppress alternate complement pathway activation. Our data suggest that these RPE defenses are weakened by genetic defects in complement pathway proteins (e.g., CFH, C/C, rs1061170) and by environmental changes that increase C3a and C5a levels around RPE cells—similar to CC-HS stimulation used in our model here. Overt activation of C3aR1 and C5aR1, as it happens by CC-HS treatment in our model or slowly as it may happen through tick-over activation of C3a and C5a around RPE cells with age, suppresses the expression of most of these complement inhibitors in RPE cells (Supplementary Fig. 12). The reduced expression of complement inhibitors, combined with lower CFH secretion on the apical side (Supplementary Fig. 3a) further weakens RPE defense mechanism and leads to a vicious cycle of RPE atrophy. Combining these reports with our data, we suggest that uncontrolled complement activation triggered over time by age-associated changes in the retina, the RPE, and the choroid slowly lead to AMD initiating events such as sub-RPE deposits, loss of RPE polarization and functionality, and eventual RPE atrophy.

Here, we report on the role of anaphylatoxins in inducing atrophy and dedifferentiation in RPE cells. Our results are consistent with the known role of C3a and C5a in causing epithelial cell dedifferentiation in liver and cancer cells^49^. RPE dedifferentiation has been reported in AMD patients’ eyes^26,27^, and in high-fat diet-fed aged *cfh* + */−* mice^13^, linking our work to these in vivo observations. C5a depletion is sufficient to ameliorate AMD cellular phenotypes in iRPE cells. Since C5a is downstream of C3a, it suggests a dominant role for C5a–C5aR1 signaling in AMD pathogenesis. However, activation of both C3a and C5a signaling has been reported in atrophic AMD and CNV animal models^9,43^.

The drug screen presented here provides a proof-of-concept that an iPSC-based platform can be used for AMD drug discovery. This drug screen design was based on calcium signaling defect-induced RPE cell death and it used clinically tested drugs. It allowed us to discover two pre-tested drugs with different mechanism of actions but robust activity in suppressing disease phenotype in CC-HS challenged RPE cells, halting disease progression and restoring tissue functionality. The apparent difference in efficacy of the two drugs on lipid deposits vs APOE deposits may reflect a more dynamic turnover of lipid-only deposits as compared to APOE containing deposits. Furthermore, the similar activity of these two drugs in restoring RPE functionality in both CFH(Y/Y) and CFH(H/H)-iRPE—as measured by their effects on the NF-κB and autophagy pathways, junctional maturity, and phagocytic ability is critical as it indicates their potential to also affect photoreceptor survival and health. Overall, this data suggests that drugs are strong candidates for testing in patients at or before the RPE atrophy stage of AMD. Furthermore, repurposing of these previously used drugs will allow their faster approval for testing in AMD patients.

L745, 870 is a known antagonist of Dopamine D4 receptor (DRD4)^50^. DRD4 is highly expressed in native and iRPE cells, and its expression increases in CC-HS treated cells (Supplementary Fig. 12). Activation of dopamine receptors is associated with similar intracellular processes induced by CC-HS treatment—calcium homeostasis defects, AKT/ERK, and NF-κB activation^51,52^. L745, 870 likely acts by blocking intracellular pathways shared by DRD4 and the two complement receptors, making it an effective drug in ameliorating AMD cellular phenotypes induced by CC-HS. Despite commonality between intracellular signaling for DRD4 and complement receptors, our data support a dominant role for C3aR1 and C5aR1 over DRD4 in inducing AMD cellular phenotypes in iRPE cells because blocking C3aR1 and C5aR1 is sufficient to rescue CC-HS induced TER drop and reduce APOE-positive deposits (Fig. 3i, j). In comparison, ACA is a serine protease inhibitor known for its role to inhibit thrombin proteases^53^. Serine proteases are one of the largest classes of proteases, including most proteases upstream of C3a and C5a generation^54^ (e.g., C1s, C1r, C2, CFB, and CFD). We hypothesize that ACA blocks the activity of most of these complement proteases, thus blocking the generation of C3a and C5a. We further extend this hypothesis to suggest that in AMD, locally delivered ACA at an earlier disease stage will block the local generation of C3a and C5a around the RPE/photoreceptor interface, stop RPE atrophy and also stop muller glia activation^55^. Furthermore, our drug analysis suggests that the recently completed phase III clinical trial for an anti-CFD antibody (Lampalizumab) designed to block the formation of C3a and C5a to slow down the progression of GA lesion would have been more effective at an earlier disease stage^4,56^.

The current model provides an in-depth analysis of complement-induced phenotypic and signaling changes in RPE monolayer and highlights the ability of iRPE cells to autonomously form drusen deposits (Fig. 8a–d). One limitation of our model is that due to the lacking photoreceptor interface, we couldn’t replicate sub-retinal drusenoid deposits that share some proteins with sub-RPE drusen deposits^57^. Another limitation is that our model doesn’t address the potential disease contribution by the atrophy of the choriocapillaris that may be influenced by other AMD-risk alleles such as in the ARMS2/HTRA1 locus^58^. CC-HS treatment is an acute model for AMD that likely conceals subtle differences between the two CFH genotypes, but this model does extend our current understanding of the role complement proteins play in inducing sub-RPE deposits and RPE atrophy. It highlights the role of activated NF-κB and downregulated autophagy in regulating AMD cellular phenotypes and suggests that drugs inhibiting NK-κB activation and autophagy downregulation may have the potential to work as anti-AMD therapeutics. Furthermore, the ability of two different drugs to inhibit several hallmarks of AMD pathology in both CFH(Y/Y) and CFH(H/H) RPE cells downstream of DRD4 or via complement inhibition further validates the translational relevance of our model. In future, we plan to perform pharmacology and pharmacokinetics of these two drugs with the goal to start a phase I clinical trial testing the tolerability and safety of these two drugs in patients.

## Methods

### Study design

This study was designed to develop an AMD model for drug discovery and drug testing. To confirm the reproducibility of this model, it was tested with three iRPE homozygous for CFH(Y/Y) variant that confers low genetic risk for AMD, four iRPE homozygous for CFH(H/H) variant that confers high genetic risk for AMD and three iRPE heterozygous for CFH(Y/H) variant that confers intermediate genetic risk for AMD (Supplementary Table 1 and Table 1). Most experiments, including drug validation use CFH(H/H) and CFH(Y/Y)-iRPE. This was done to ensure that there is no genotype bias in the outcome of experiments, to discover if the high or the low-risk alleles respond differently to the complement insult, and to confirm that drugs work on broad AMD genetics. Drug screening was performed using a commercially available library—LOPAC (LO4200, Sigma).

iPSCs work and patient sample collection were approved by the Combined NeuroScience Institutional Review Board (CNS IRB) under the Office of Human Research Protection (OHRP), NIH as per 45 CFR 46 guidelines of U.S. Government. Patient samples were collected using CNS IRB-approved consent form in accordance to the criteria set by the Declaration of Helsinki under the protocol number NCT01432847 (https://clinicaltrials.gov/ct2/show/NCT01432847?cond=NCT01432847&draw=2&rank=1). The use of cadaver AMD eyes was exempt from the NIH Institutional Review Board approval as per 45 CFR 46 guidelines of U.S. Government.

### iPSC lines, iPSC-RPE differentiation, and functional validation

The iPSC lines used in the study are listed in Supplementary Table 1 and Table 1. For iPSC genotype, TaqMan genotyping assays (ThermoFisher) were used. The following probes were tested: rs1061170 (Assay ID: AHI1TPW;4331349); rs2230199 (Assay ID: C__26330755_10;4351379). SNP rs10490924 (Assay ID: C__29934973_20;4351379).

For differentiation iPSCs were seeded onto vitronectin (A1700, ThermoFisher)-coated six-well plates. After 2 days in E8 media (A1517001, ThermoFisher) supplemented with Rock Inhibitor (1254, Tocris Bioscience), iPSC colonies formed a confluent monolayer, and were transitioned to the differentiation media ((DMEM/F12 (11330032, ThermoFisher), N2 supplement (A1370701, ThermoFisher), B27 (17504044, ThermoFisher), KSR (12618013, ThermoFisher), 20 ng/ml NOGGIN (6057 R&D Systems), 5 μM CK1-7 Dihydrochloride (C0742, Sigma), 5 μM SB 431542 hydrate (S4317, Sigma), and 5 ng/ml IGF-1 (AFL291, R&D Systems), 5 μM PD0325901 (PZ0612, Sigma), 10 mM nicotinamide (N0636, Sigma), 150 ng/ml ACTIVIN A (338-AC/CF, R&D Systems)). Cells that reached the RPE committed phase of differentiation were reseeded onto fresh vitronectin coated surfaces and maintained in RPE maintenance media (RPEMM) (MEM + glutamax, 32561037, ThermoFisher; 5%FBS, SH30071.03, Hyclone; Taurine, T-0625, Sigma; Thyronine, T-5516, Sigma; Hydrocortisone, H-0396-10, Sigma) for 15 days. RPE cells enriched with anti-CD24 (1:500, 655154, BD Biosciences) and anti-CD56 (1:500, 340723, BD Biosciences) antibodies, then seeded onto vitronectin coated transwells (3460, Corning), and cultured for 6 weeks before assays and experiments. All iPSC work was performed under institutional review board-approved protocol #11-E1-0245. All iPSC-RPE cultures were quality controlled before use and no cultures below a TER cut-off 400 Ohms cm^2^ were used.

### Human sera and drug treatments

In all, 6–8 weeks old fully matured, polarized iRPE monolayers were used for all the experiments. iRPE cells were treated for 48 h with either 5% CC-HS (NHS, Complement technology; ISERABH, Innovative research; S1-LITER, EMD Millipore; CC-HS from all three companies showed comparable activity), or 5% CI-HS (heat-inactivated CC-HS), or 5% C5-depleted serum (A320, Complement technology), or 5% C3-depleted serum (A314, Complement technology), or 5% CFD depleted serum (A336, Complement technology) supplemented in RPEMM, with daily medium change. For the receptor blocker experiments, iRPE cells were treated with 10 μM PMX53 (5453, Tocris), and 10 μM compstatin (2585, Tocris) for 48 h in 5%CC-HS. For drugs experiments, 30 μM aminocaproic acid (ACA) (A2504, Sigma), or 6 μM L745,870 (L745) (1002, Tocris), iRPE monolayers were treated in the following ways: (1) iRPE cells were treated for 24 h with ACA or L745 in 5% RPEMM media followed by 48 h with ACA or L745 along with CC-HS; (2) iRPE cells were treated with CC-HS for 24 h followed by 168 h (7 days) with ACA or L745 in 5% RPEMM media; (3) iRPE cells were treated for 24 h with 5% CC-HS followed by 24 h with ACA, or L745 in 5% RPEMM media.

iRPE monolayers were fixed in 4% paraformaldehyde for 20 m, at RT. The cells or membranes (for APOE immunostaining, iRPE cells were stripped from the transwell membrane by 10-m incubation in deionized (DI) water) were washed three times with 1× phosphate buffered saline (PBS), and blocked in Immunocytochemistry (ICC) buffer (1× PBS, 10010-023, ThermoFisher), 1% bovine serum albumin (BSA) (160069, MP Biomedicals), 0.25% Tween20 (900-64-5, Affymetrix), 0.25% Triton X-100 (9002-64-5, Sigma) for 1 h at RT. Primary antibodies except for APOE (1:1000) were diluted, 1:100, in ICC buffer, added to the cells or membranes and incubated overnight at RT. Primary antibodies against the following proteins were used: p65 (2 µg/mL; 8242, CST), ATG5 (2 µg/mL; 12994, CST), ATG7 (0.4 µg/mL; 8558, CST), LC3 (0.5 µg/mL; 2775, CST), APOE (~250 ng/mL; AB947, Millipore), ZO-1 (5 µg/mL; MA3-39100-A488, ThermoFisher), EZRIN (1 µg/mL; E8897, Sigma), COLLAGEN IV (~5 µg/mL; ab6311, Abcam), C5aR1 (~1 µg/mL; ab11867, Abcam), C3aR1 (~1 µg/mL; 126250, Abcam), RELB (~2 µg/mL; 4922, CST), TRAF3 (190 ng/mL; 4729, CST), VIMENTIN (~10 µg/mL; ab92547, Abcam), CLDN19 (~10 µg/mL; ab74374, Abcam), C5b-9 (5 µg/mL; M0777, Dako), β-CATENIN (12 µg/mL; C7738, Sigma), Na^+^ K^+^ ATPase (1 µg/mL; ab76020, Abcam). Following the overnight primary antibody incubation, the cells or membranes were washed three times with ICC buffer. Secondary antibodies were diluted 1:1000 in ICC buffer, added to the cells or membranes, and incubated in dark for one hour at RT. Secondary antibodies use: Alexa Fluor 555 donkey anti-rabbit (5 µg/mL; A31572, ThermoFisher), Alexa Fluor 488 donkey anti-rabbit (1:1000) (5 µg/mL; A21206, ThermoFisher), Alexa Fluor 555 donkey anti-mouse (1:1000) (5 µg/mL; A31570, ThermoFisher), Alexa Fluor 488 donkey anti-mouse (1:1000) (5 µg/mL; A21202, ThermoFisher), or Alexa Fluor 555 donkey anti-goat (5 µg/mL; A32816, ThermoFisher), Hoechst-33542 (H3570, 1:2000, ThermoFisher), or—for specific experiments—Phalloidin (Alexa Fluor™ 488, 1:300, A12379, ThermoFisher). The cells and membranes were washed three times with ICC buffer, and mounted on a glass slide, with Fluoromount-G aqueous mounting medium (0100-01; Southern tech), and a glass coverslip. Samples were imaged using Zeiss 880 (×63, NA1.4 oil) or Zeiss 700 (×63, NA1.4 and ×20, NA 0.5) or Zeiss 800 (×63, 1.4 and ×25, NA 0.8) confocal microscopes (Carl Zeiss). Images were processed and exported as tiff files using Zenblue3.2 software (Carl Zeiss).

Lipid deposits were stained using the Lipid Oil Red O kit (MAK194-1KT, Sigma). The cells were fixed with 10% formalin for 30 m to an hour at RT, then washed three times with DI water. 60% isopropanol was gently added to the cell surface and incubated for 5 m at RT. Isopropanol was aspirated and the cells were incubated in the Oil Red O working solution (three parts of Oil Red O stock to two parts isopropanol) for 30 m at RT; then washed three times with DI water. Hematoxylin was added to the cell surface for 5 m at RT and washed with DI water until all excess dye was removed^59^. The cells were mounted and imaged as described above. For Nile red staining, the cells were fixed in 4% paraformaldehyde for 1 h at RT and washed three times with 1× PBS. The Nile red (72485, Sigma) stock solution is prepared in acetone with a concentration of 3 mg/ml, then diluted 1:500 in 1× PBS to generate the working solution. Both the stock and working Nile red solutions were incubated for 20 m at RT before filtering with a 0.22-μm filter. Cells were incubated in the Nile red working solution for 30 m at RT, washed three times with 1× PBS, mounted, and imaged using the procedure described above. Quantification of APOE and lipid droplets (Nile Red and BODIPY) was performed in ImageJ (v1.8.0, Bethesda, USA). For APOE, particles were counted with the following parameters—internodes thresholding, size 25–400 µm^2^, circularity 0–1. For Nile Red and BODIPY- triangle thresholding, size 0.01–400 µm^2^ and circularity 0–1 were used. The number of images and cell line information is listed in figure legends.

### Transmission electron microscopy

iRPE monolayers were fixed overnight in 2.5% glutaraldehyde (G6257, Sigma Aldrich), washed three times with 1× PBS (10010-023, ThermoFisher), and treated with 1% ice-cold osmium tetroxide (75633, Sigma Aldrich) in 1× PBS solution for 1 h. The cells were then rinsed in 1× PBS and placed through stepwise ethanol dehydration steps (three washes in 50%, 70%, 85%, and 100% ethanol; 50980462, Fisher Sciences) prior to embedding. Ultrathin sections (~50–90 nm thick) were cut on an ultramicrotome and mounted on Pelco copper grids (IGC50; Ted Pella, Inc.). TEM images were acquired on a JEOL JEM-1010 transmission electron microscope (JEOL, Peabody, MA).

### Scanning electron microscopy

iRPE cells were stripped from the transwell membrane by 10-min incubation in deionized (DI) water) were washed three times with 1× PBS before fixing the transwell membrane in 2.5% glutaraldehyde + 1% formaldehyde in 0.1 M sodium cacodylate buffered solution (16537-06, Electron Microscopy Sciences). Samples were cut to fit the sample holder and underwent sequential ethanol (50980462, Fisher Sciences) dehydration: ddH20 (5 min), 30% ethanol (10 min), 50% ethanol (10 min), 70% ethanol (20 min), 90% ethanol (5 min), 95% ethanol (5 min), 100% ethanol (5 min), 100% ethanol (5 min), 100% ethanol (5–20 min) while transported to the critical point drying (CPD) machine (Leica EM CPD300). The CPD was operated as per the manufacturer’s protocol to further dehydrate to be compatible with the high vacuum required for scanning electron microscopy. The following CPD parameters were used: CO_2_ in: (speed: slow), (delay: 2 min); exchange: (speed: 1), (cycles: 16); gas out: (heat: slow), (speed: slow). Samples were then mounted onto SEM studs (16111, Ted Pella Inc) and stored in a desiccator until imaged on a Zeiss EVO MA 10 SEM.

### Transepithelial resistance, electrophysiology, and calcium recordings

Transepithelial resistance (TER) is a measure of intactness of tight junctions between neighboring cells and reflects mature monolayer tissue. Two different methods were used to measure TER across the iRPE monolayers: (1) using commercially available Epithelial Volt/Ohm Meter (EVOM2, WPI); (2) modified Ussing chamber-based electrophysiology^28^. was used.

1. EVOM2: The electrodes in the form of chopsticks (STX2, WPI) were applied on the apical and basal sides of iRPE, the current was passed, and resistance values (Ohms) were noted down for each condition. Actual resistance (Ohms.cm2) is calculated by multiplying TER to the area of measurement (12 mm diameter transwell in this case).
2. Modified Ussing Chamber: Unlike EVOM2 that can only measure monolayer TER, in our custom electrophysiology set up we can also measure monolayer transepithelial potential (TEP). TEP is the difference in resting membrane potential of apical and basal membranes of an RPE cell. It reflects the differential expression of ion channels on the two membranes. A higher TEP suggests higher functional polarization and a fully mature RPE monolayer. For TEP measurements, iRPE cells seeded on transwell were mounted in a modified Ussing chamber. Calomel electrodes were used in Ringer’s solutions and Agar bridges to measure the transepithelial potential (TEP)^29^. The signals from intracellular microelectrodes were referenced to the basal bath, to measure the basolateral membrane potential (Vb); while the apical membrane potential (Va) was calculated by the equation: Va = Vb-TEP. The total transepithelial resistance (TER) and the ratio of the apical to basolateral membrane resistance (R_a_/R_b_), were obtained by passing 2–4 mA current pulses across the tissue, and measuring the resultant changes in TEP, Va, and Vb. For the determination of intracellular calcium levels, a ratiometeric dye, Fura-2 (F1221, ThermoFisher) was used. CI-HS and CC-HS treated cells were incubated with 33 μM FURA2 and 0.007% Pluronic (P3000MP, ThermoFisher Scientific) at RT for an hour. To record change in intracellular Ca^2+^ levels in response to ATP a polychrome V monochromator (TILL Photonics; FEI Life Sciences) was used in conjunction with custom software written in LabVIEW to provide high-speed photic excitation between 340 and 385 nm every 0.5 s and a photomultiplier tube measuring the emission fluorescence at 510 nm.

### Phagocytosis assay

iRPE cells were fed with pHrodo^TM^ (A10026, ThermoFisher) labeled bovine photoreceptor outer segments (POS) (98740, InVision Bioresources) 10 POS/iRPE cell, for 4 h. Untreated cells were used as a control. After 4 h, cells were washed four times with 1× PBS (10010-023, ThermoFisher), and incubated in 0.25% Trypsin-EDTA (25200056, ThermoFisher) for 20 min. The 0.25% Trypsin-EDTA was aspirated off and replaced with RPEMM. Cells were collected in a tube and centrifuged at 200 × *g* for 5 min. Cell pellets were washed three times with 1× PBS. After the final wash, pellets were resuspended in 600 μL of 0.1%BSA in 1× PBS; cell suspension was strained through a 40-μm cell strainer (14-959-49 A, BD Biosciences). In total, 5 μg/mL of DAPI (D1306, ThermoFisher) was added to each tube that contained cell suspension. pHrodo^TM^ signal (mean fluorescence intensity) from phagocytosed POS was measured by MACSQuant^®^ Analyzers (MACS Miltenyi Biotech) for a total of 10,000 counts/treatment. Data were analyzed by FlowJo software. The ratio of labeled POS fed to dye-fed samples was plotted.

### Real-time PCR

Total RNA from iRPE cells was extracted using the NucleoSpin RNA kit (740955; Machery-Nagel), as per the manufacturer’s protocol. The concentration of the collected RNA was quantified using an ND1000 spectrophotometer (Nanodrop Technologies). cDNA from isolated RNA was synthesized using the iScript™ cDNA Synthesis Kit (1708891, Bio-Rad), per the manufacturer’s protocol. Each reaction in the gene expression analysis used 10 ng of cDNA and 5 μL of SsoAdvanced™ Universal SYBR^®^ Green Supermix (1725274, Bio-Rad). Gene array plates for autophagy (10034452, Bio-Rad) and NF-kB (10034545, Bio-Rad) pathways from Bio-Rad were used with a Viia7 Real-Time PCR System (ThermoFisher Scientific).

### Western blotting

iRPE cells were lysed and collected from transwell membranes with RIPA buffer (89900, ThermoFisher), then centrifuged at 4 **°**C and 15,989× *g* for 10 m. BCA protein assay (23227, ThermoFisher) was carried out, as per the manufacture’s protocol, to check the concentration of all of the iRPE cell lysates. SDS-PAGE for western blot assays were carried out using 4–16% pre-made gels (AnykD™ Criterion™ TGX™ Precast Midi Protein Gel, 12 + 2 well, 45 µl, cat #5671123, Bio-Rad), and all samples were loaded at equal protein levels (40–80 µg/well). A semi-dry transfer method was carried out using a TransBlot Turbo transfer system (1704150, Bio-Rad). The blots were blocked in 5% BSA buffer 5% BSA + PBST (1× PBS and 0.5% Tween20) for 1 h at RT. The primary antibody of interest was diluted 1:1000 in 5% BSA buffer, added to the blots, and incubated overnight at 4 °C on a shaker. The primary antibody solution was decanted, and the blots were washed three times with PBST. The primary antibodies against the following were used: LC3B (detects both LC3-I and LC3-II^60^; 52 ng/mL, 2775, CST), ATG5 (220 ng/mL, 12994, CST), ATG7 (41 ng/mL, 8558,CST), Total ERK1/2 (251 ng/mL, 4696, CST), p-ERK1/2 (191 ng/mL, 9101S, CST), Total AKT (35 ng/mL, 4691S, CST), pAKT Ser 473 (91 ng/mL, 4060, CST), and β-ACTIN (diluted 1:3000) (208 ng/mL, 3700S, mouse), 370 ng/mL, 8457 S, rabbit, CST). Secondary antibodies—goat anti-rabbit HRP-conjugate (1705046, Bio-Rad) and goat anti-mouse HRP-conjugate (1705047, Bio-Rad), were diluted (1:5000) in 5% BSA buffer, added to the blots, and incubated for an hour in the dark at RT. Secondary solutions were removed and blots were washed three times with PBST, and imaged using a Bio-Rad imaging machine. The quantification of all blots was performed using Image lab software (version 2.3.0.07, Bio-Rad), and the results are presented as the ratio of protein of interest to β-ACTIN.

### IL-8, IL-18, CFH, C3a, and C5a ELISAs from supernatants of cells

iRPE on transwells were treated with CI-HS or CC-HS for 48 h. Media from the apical and basal sides of the transwells were collected in 1.5-mL Eppendorf tubes and centrifuged at 17,243 × *g* and 4 °C for 15 m. The supernatants were transferred into fresh 1.5-mL Eppendorf tubes, immediately placed in dry ice, and stored at −80 °C. ELISA kits for cytokines IL-8 (LXSAHM-02, R&D) and IL-18 (LXSAHM-02, R&D) were used per the manufacturer’s protocol. Samples were diluted fivefold in the diluent buffer. ELISA kits for complement proteins CFH (A039, Quidel), C3a (A031, Quidel), and C5a (A021, Quidel) were used per the manufacture’s protocol. Samples for the CFH and C3a assays were diluted 50-fold, and samples for the C5a assay were diluted fivefold. ELISA plates were analyzed using a TECAN SAFIRE plate reader. Results account for different apical and basal media volumes used for transwells.

### RNA isolation and RNAseq analysis

RNA was isolated from RPE monolayer 8 weeks post seeding on transwells, using RNeasy Kit (Qiagen, Germantown, MD, USA). Quantification and RNA integrity were assessed using nanodrop and bioanalyzer, respectively. Poly(A)-enriched mRNA sequencing was performed on the Illumina platform. RNA-Seq libraries were constructed from 1 µg total RNA using the Illumina TruSeq^®^ RNA Sample Prep Kits, version 2 which includes poly-A enrichment. After second-strand synthesis, the cDNA was fragmented using the Covaris E210 (26593, American Laboratory Trading). Library amplification was performed using ten cycles to minimize the risk of over-amplification. Unique single-index barcode adapters were applied to each library. Libraries were pooled in equimolar ratio and sequenced together on a HiSeq 4000. At least 46 million 75-base read pairs were generated for each individual library. Data were processed using RTA 2.7.7 and CASAVA 1.8.2. Gene- and transcript-level expression was quantified with quasi-mapping (Salmon) and differential expression was calculated with DESeq2.

The Salmon tool (with the–seqBias and–gcBias corrections applied) was used to quantify transcript expression using Gencode (version 27). The transcript-level quantification was collapsed to the gene level with the tximport tool in R (3). The gene differential expression analysis was conducted with the DESeq2 tool, where we removed the batch effects from cell line-specific variation by setting it as a covariate. For the heatmap visualization, the Euclidean distance between samples was calculated in R with the included dist function. The heatmaps were plotted with the Complex Heatmap tool. For the principal component (PC) analysis, the R function prcomp was used to calculate the distances between the samples^61^.

### Primary high-throughput screen

iRPE4 was used for the primary screen. A previously published reporter iPSC line (iRPE4) which expresses green fluorescent protein (GFP) under the control of an RPE-specific tyrosinase enhancer was used for the primary screen^23^. GFP signal was used to ensure iRPE maturity in 384-well plates^23^. A laser scanning cytometer, Acumen eX3 (TTP Labtech) was used to read the intracellular GFP signal. iRPE4 cells were cultured in 40 μL of RPEMM in a black clear bottom 384-well plates (3542, Corning) for 28 days. The medium was aspirated and 10 μM of A23187 in 40 μL of phenol red-free RPEMM (41061029, ThermoFisher) was dispensed into the 384-well plates using a Multidrop™ Combi Reagent Dispenser (5840300, ThermoFisher). Compounds from LOPAC (LO1280-1KT, Sigma) library were immediately dispensed using an acoustic dispensing system at final concentrations of 46 and 9.2 μM. Fluorescence from GFP expressing cells was quantitated with an Acumen laser scanning imaging cytometer (TTP Labtech Inc, Cambridge, MA). The Acumen software was used to measure the total number and intensity of fluorescent objects. Following the read for fluorescence, 40 μL of CellTiter Glo (G7570, Promega), which measures intracellular ATP, was added to the assay plates using a Multidrop™ Combi Reagent Dispenser (5840300, ThermoFisher). Luminescence was measured on the Viewlux (Perkin Elmer). The GFP signal intensity measured for each well was normalized to the median fluorescence intensity per cell from the DMSO control wells as 100% signal and fluorescence intensity per cell from control wells with A23187 at an EC_100_ as 0% signal. For the cell proliferation assay, relative luminescence units (RLU) for each well were normalized to the median RLUs from the DMSO control wells as 100% viability, and median RLUs A23187 at an EC_100_ as 0% viability. The activity of the hits from the primary screen was determined based on two parameters: a) Viability at the lower dose (C2 = 9.2 μM) being >40% and Viability at the higher dose (C1 = 46 μM) being > −10% b) Viability of >30% at the higher dose.

### Secondary high-throughput screen

iRPE1 and 4 were cultured in RPEMM for 28 days in 384-well plates. Cells were seeded at a density of 5000 cells/well using a Multidrop™ Combi Reagent Dispenser (5840300; ThermoFisher). For each 384-well plate, the negative control was DMSO alone, added at 0.5% and the positive control was A23187 co-dosed with DMSO. Drug-treated cells were co-dosed with 2.5 μM or 5 μM or 10 μM of A23187. In total, 45 selected compounds from the primary screen were used in this secondary screen at seven doses ranging from 0.78 to 50 μM. Cells were incubated for 48 h, and cell viability was measured with CellTiter-Glo reagent (Promega) on the Viewlux which measures intracellular ATP. Normalized data ere fitted to a 4-parameter dose response curves using a custom grid-based algorithm to generate curve response class (CRC) score for each compound dose response. Since the primary screen was done at two concentrations, dose response curves were only generated for the secondary screen data.

### Human donor eye

Human cadaver eyes were obtained through the Advancing Sight Network (Alabama Eye Bank). AMD and non-AMD diagnoses were based on donors’ medical records. Death-to-preservation time was 3.3 h for the non-AMD eye and 3.6 h for the AMD eye. Complement receptor immunostaining was performed on a healthy eye from a 67-year-old male donor with no history of retinal degenerative diseases. VIMENTIN (~10 µg/mL, Ab92547, 1:100, Abcam) and Phalloidin (A12379, 1:300, ThermoFisher) immunostaining staining was performed on an AMD eye from a 98-year-old female donor with dry-AMD diagnosis. Eyes were fixed in 4% paraformaldehyde (15949, Electron Microscopy Sciences) for 4 h at room temperature, the retina was detached from RPE to perform the RPE-choroid wholemount immunostaining.

### Graph plotting and statistical methods

The data were presented as boxplots, where the box limits represent the first and third quartile, the centerline shows the median and the whiskers indicate the 5th and 95th percentiles so that the range specifies 90% of the data. When two independent groups were compared, Welch’s *t* test was used. Dunnett’s test post hoc pairwise multiple comparisons procedure was used to compute statistics comparing several treatment groups relative to the control including Bonferroni correction. For both *t* test and Dunnett’s test, *P* values < 0.05 were considered significant. Shapiro–Wilk, *t* test and ANOVA were performed in base R (version 3.5.1)^62^, while Dunnett’s test was calculated using DescTools package for R. The R packages dplyr^63^ and ggplot2^64^ were used for data handling and plotting, respectively.

### Reporting summary

Further information on research design is available in the Nature Research Reporting Summary linked to this article.

## Supplementary information

Supplementary Information

Reporting Summary

## Data Availability

The bulk RNAseq data generated in this study have been deposited in the GEO database under accession code GSE185310. These RNAseq data are openly available without any restriction. All the processed data are available within the article. All the raw data generated in this study are provided in the Supplementary Information/Source Data file. Source data are provided with this paper.

## Source data

Source Data
