# Supplementary material for: Epithelial phenotype restoring drugs suppress macular degeneration phenotypes in an iPSC model
Source: Nat Commun. 2021 Dec 15;12:7293. doi: 10.1038/s41467-021-27488-x (PMC8674335; doi:10.1038/s41467-021-27488-x)
Supplement: Supplementary file 2 — Reporting Summary [file 41467_2021_27488_MOESM2_ESM.pdf]

nature portfolio

Corresponding author(s): Bharti, Kapil
NCOMMS-19-42025

Last updated by author(s): Oct 29, 2021

Reporting Summary

Nature Portfolio wishes to improve the reproducibility of the work that we publish. This form provides structure for consistency and transparency in reporting. For further information on Nature Portfolio policies, see our Editorial Policies and the Editorial Policy Checklist.

Statistics

For all statistical analyses, confirm that the following items are present in the figure legend, table legend, main text, or Methods section.

n/a	Confirmed	
☐	☒	The exact sample size (*n*) for each experimental group/condition, given as a discrete number and unit of measurement
☐	☒	A statement on whether measurements were taken from distinct samples or whether the same sample was measured repeatedly
☐	☒	The statistical test(s) used AND whether they are one- or two-sided<br>*Only common tests should be described solely by name; describe more complex techniques in the Methods section.*
☐	☒	A description of all covariates tested
☒	☐	A description of any assumptions or corrections, such as tests of normality and adjustment for multiple comparisons
☐	☒	A full description of the statistical parameters including central tendency (e.g. means) or other basic estimates (e.g. regression coefficient) AND variation (e.g. standard deviation) or associated estimates of uncertainty (e.g. confidence intervals)
☐	☒	For null hypothesis testing, the test statistic (e.g. *F*, *t*, *r*) with confidence intervals, effect sizes, degrees of freedom and *P* value noted<br>*Give P values as exact values whenever suitable.*
☒	☐	For Bayesian analysis, information on the choice of priors and Markov chain Monte Carlo settings
☒	☐	For hierarchical and complex designs, identification of the appropriate level for tests and full reporting of outcomes
☒	☐	Estimates of effect sizes (e.g. Cohen's *d*, Pearson's *r*), indicating how they were calculated

Our web collection on statistics for biologists contains articles on many of the points above.

Software and code

Policy information about availability of computer code

Data collection	All imaging based data was collected using commercial Zenblue3.2 software from Zeiss
Data analysis	All images were analyzed using Image J (v1.8.0,Bethesda, USA). An open source R-Software (v3.5.1.) a commercial software graph pad Prism 9.1.0, and an open-source Cluster Profiler version 3.4.4 were used for data analysis. Biorad software Image Lab version 2.3.0.07 was used for quantification of Western blots. RNAseq data was processed using RTA 2.7.7 and CASAVA 1.8.2. Transcript expression was quantified using Gencode (version 27) and R(v3.5.1).The gene differential expression analysis was conducted with the DESeq2 tool (v2).

For manuscripts utilizing custom algorithms or software that are central to the research but not yet described in published literature, software must be made available to editors and reviewers. We strongly encourage code deposition in a community repository (e.g. GitHub). See the Nature Portfolio guidelines for submitting code & software for further information.

Data

Policy information about availability of data

All manuscripts must include a data availability statement. This statement should provide the following information, where applicable:

- Accession codes, unique identifiers, or web links for publicly available datasets
- A description of any restrictions on data availability
- For clinical datasets or third party data, please ensure that the statement adheres to our policy

The bulk RNAseq data generated in this study have been deposited in the GEO database under accession code GSE185310 [https://www.ncbi.nlm.nih.gov/geo/query/acc.cgi?acc=GSE185310]. This RNAseq data are openly available without any restriction. All the processed data are available within the article. All the raw data generated in this study are provided in the Supplementary Information/Source Data file.

Field-specific reporting

Please select the one below that is the best fit for your research. If you are not sure, read the appropriate sections before making your selection.

☒ Life sciences ☐ Behavioural & social sciences ☐ Ecological, evolutionary & environmental sciences

For a reference copy of the document with all sections, see nature.com/documents/nr-reporting-summary-flat.pdf

Life sciences study design

All studies must disclose on these points even when the disclosure is negative.

Sample size	Statistical analysis was performed using two-way ANOVA or unpaired t-test. Both of these methods require a minimum of three samples in each treatment group. For that reason, it was ensured to have minimum of three samples in each treatment group.
Data exclusions	No data was excluded from the analysis
Replication	All experiments were replicated at least three (often more) independent times, by two different authors of the paper. For each figure, the number of independent experiments or biological replicates is indicated in the figure legends.
Randomization	Cells in a given experiment were randomly assigned to treated and untreated groups and treated and untreated groups were randomly analyzed
Blinding	Blinding was performed during the treatment of different groups. One operator prepared cell culture medium with CIHS and CCHS treatments and another operator treated samples with different medium. CIHS and CCS groups were treated equally. For analysis, sample names were masked when possible and analysis was performed using objective quantitative methods.

Reporting for specific materials, systems and methods

We require information from authors about some types of materials, experimental systems and methods used in many studies. Here, indicate whether each material, system or method listed is relevant to your study. If you are not sure if a list item applies to your research, read the appropriate section before selecting a response.

Materials & experimental systems

n/a	Involved in the study	
☐	☒	Antibodies
☐	☒	Eukaryotic cell lines
☒	☐	Palaeontology and archaeology
☒	☐	Animals and other organisms
☐	☒	Human research participants
☒	☐	Clinical data
☒	☐	Dual use research of concern

Methods

n/a	Involved in the study	
☒	☐	ChIP-seq
☒	☐	Flow cytometry
☒	☐	MRI-based neuroimaging

Antibodies

Antibodies used	LC3B (detects both LC3-I and LC3-II61; cat#2775, Cell Signaling Technology), ATG5 (cat #12994, Cell Signaling Technology), ATG7 (cat#8558,Cell Signaling Technology), Total ERK1/2 (cat#4696, Cell Signaling Technology), p-ERK1/2 (cat#9101S, Cell Signaling Technology), Total AKT (cat#4691S, Cell Signaling Technology), p-AKT Ser 473 (cat#4060, Cell Signaling Technology), and β-ACTIN (diluted 1:3000) (cat#3700S, mouse), 8457S, rabbit, Cell Signaling Technology). Secondary antibodies—Goat Anti-Rabbit HRP conjugate (cat#1705046, Bio-Rad) and Goat Anti-Mouse HRP conjugate (cat#1705047, Bio-Rad), were diluted (1:5000).p65 (cat#8242, Cell Signaling Technology), ATG5 (cat#12994, Cell Signaling Technology), ATG7 (cat#8558, Cell Signaling Technology), LC3 (cat#2775, Cell Signaling Technology), APOE (cat#AB947, Millipore), ZO-1 (cat#MA3-39100-A488, ThermoFisher), EZRIN (cat#E8897, Sigma), COLLAGEN IV (cat#ab6311, Abcam), C5aR1 (cat#ab11867, Abcam), C3aR1 (cat#126250, Abcam), RELB (cat#4922, Cell Signaling Technology), TRAF3 (cat#4729, Cell Signaling Technology), VIMENTIN (cat#ab92547, Abcam), CLDN19 (cat#ab74374,Abcam), C5b-9 (cat#M0777, Dako), β-CATENIN (cat#C7738, Sigma), Na+K+ATPASE (cat#ab76020, Abcam), FIBULIN 3 (cat#PA5-34788, ThermoFisher), CD24(Cat#655154, BD Biosciences), CD56 (1:500cat#340723, BD Biosciences)
Validation	All primary antibodies used in this study were commercially available were either validated by the manufacturer for the particular application or cited in the literature.<br>LLC3B (detects both LC3-I and LC3-II61; cat#2775, lot 13, CST) https://www.cellsignal.com/products/primary-antibodies/lc3b-antibody/2775. Citations- IF- PMID: 32724374,31752345, WB- PMID: 33182483, 31752345<br>ATG5 (cat #12994, lot 5, clone D5F5U, CST) https://www.cellsignal.com/products/primary-antibodies/atg5-d5f5u-rabbit-mab/12994 Citations IF PMID: 32677505 30327467, 31070476, WB PMID: 31519908<br>ATG7 (cat#8558, lot 4, clone D12B11, CST) https://www.cellsignal.com/products/primary-antibodies/atg7-d12b11-rabbit-mab/8558?_=1634910975357&Ntt=8558&tahead=true Citations IF PMID: 33226137 WB PMID: 31519908

Total ERK1/2 (cat#4696, lot 29, clone L34F12, CST), https://www.cellsignal.com/products/primary-antibodies/p44-42-mapk-erk1-2-l34f12-mouse-mab/4696 Citations WB PMID: 33215223

p-ERK1/2 (cat#9101S, lot 31, CST) https://www.cellsignal.com/products/primary-antibodies/phospho-p44-42-mapk-erk1-2-thr202-tyr204-antibody/9101?site-search-type=Products&N=4294956287&Ntt=9101s&fromPage=plp&_requestid=2576813 Citations WB PMID: 33685316

Total AKT (cat#4691S, lot 28, clone C67E7, CST), https://www.cellsignal.com/products/primary-antibodies/akt-pan-c67e7-rabbit-mab/4691 Citations WB PMID: 32164618

p-AKT Ser 473 (cat#4060, lot 26, clone D9E, CST), https://www.cellsignal.com/products/primary-antibodies/phospho-akt-ser473-d9e-xp-rabbit-mab/4060 Citations WB PMID: 33509092

β-ACTIN (diluted 1:3000) (cat#3700S, lot 20, clone 8H10D10, mouse)(cat#8457S, lot 8, clone D6A8, rabbit, CST), https://www.cellsignal.com/products/primary-antibodies/b-actin-8h10d10-mouse-mab/3700?site-search-type=Products&N=4294956287&Ntt=3700s&fromPage=plp&_requestid=2579004

https://www.cellsignal.com/products/primary-antibodies/b-actin-d6a8-rabbit-mab/8457 Citations WB PMID: 33149810, PMID: 32945446

p65 (cat#8242, lot 16, clone D14E12, CST) https://www.cellsignal.com/products/primary-antibodies/nf-kb-p65-d14e12-xp-rabbit-mab/8242 Citations IF PMID: 31391462

APOE (cat#AB947, lot 3524799, Millipore) https://www.emdmillipore.com/US/en/product/Anti-Apolipoprotein-E-Antibody,MM_NF-AB947 Citations IF PMID: 21969589

ZO-1 (cat#MA3-39100-A488, lot WE327289, clone ZO1-1A12, ThermoFisher) https://www.thermofisher.com/antibody/product/ZO-1-Antibody-clone-ZO1-1A12-Monoclonal/MA3-39100-A488 Citations IF PMID: 31714897 WB

EZRIN (cat#E8897, lot 039M4838V, clone 3C12, Sigma) https://www.sigmaaldrich.com/US/en/product/sigma/e8897 Citations IF PMID: 31714897 WB

COLLAGEN IV (cat#ab6311, lot GR3298256-5(1 000mg/ml), clone COL-94, Abcam), https://www.abcam.com/collagen-iv-antibody-col-94-ab6311.html Citations IF PMID: 30651323, WB

C5aR1 (cat#ab11867, clone S5/1 Abcam), https://www.abcam.com/c5a-r-antibody-s51-ab11867.html

Validation used positive controls in the assay for both IF and WB . Citations IF PMID: 30651323, WB

C3aR1 (cat#126250, Abcam) https://www.abcam.com/c3a-r-antibody-ab126250.html

Validation used positive controls in the assay for both IF and WB. Citations IF, WB PMID: 34066088

RELB (cat#4922, lot 3, clone C1E4, CST), https://www.cellsignal.com/products/primary-antibodies/relb-c1e4-rabbit-mab/4922 Citations IF PMID: 29074539

TRAF3 (cat#4729, lot 3, CST) https://www.cellsignal.com/products/primary-antibodies/traf3-antibody/4729 Citations IF PMID: 26882989,

VIMENTIN (cat#ab92547, lot GR3258719-4(0.268mg/mL)YP111523PS1, clone EPR3776, Abcam) https://www.abcam.com/vimentin-antibody-epr3776-cytoskeleton-marker-ab92547.html Citations IF PMID: 33670907,

CLDN19 (cat#ab74374, Abcam), https://www.abcam.com/cldn19-antibody-ab74374.html

C5b-9 (cat#M0777, clone aE11, Dako), https://www.agilent.com/en/product/immunohistochemistry/antibodies-controls/primary-antibodies/c5b-9-(concentrate)-153988 Citations IF PMID: 21969589

β-CATENIN (cat#C7738, lot 0000084485, clone 15B8, Sigma). https://www.sigmaaldrich.com/US/en/product/sigma/c7738 Citations IF PMID: 29298421

Na+K+ATPASE (cat#ab76020, lot GR3184452-3, clone EP1845Y, Abcam), https://www.abcam.com/sodium-potassium-atpase-antibody-ep1845y-plasma-membrane-loading-control-ab76020.html Citations PMID: 33069918

CD24(Cat#655154, clone ML5, BD Biosciences), https://www.bdbiosciences.com/en-us/products/reagents/flow-cytometry-reagents/clinical-diagnostics/single-color-antibodies-asr-ivd-ce-ivd/cd24-fitc.655154 Validation- validated for MACS enrichment, PMID: 30651323

CD56 (1:500cat#340723, clone NCAM16.2, BD Biosciences), PMID: 30651323

https://www.bdbiosciences.com/en-us/products/reagents/flow-cytometry-reagents/clinical-diagnostics/single-color-antibodies-asr-ivd-ce-ivd/cd56-fitc.340723 Validation- validated for MACS enrichment

Goat Anti-Rabbit HRP conjugate (cat#1705046, lot 64320357, Bio-Rad) https://www.bio-rad.com/en-us/sku/1705046-immun-star-goat-anti-rabbit-gar-hrp-conjugate?ID=1705046

Goat Anti-Mouse HRP conjugate (cat#1705047, lot 64320659, Bio-Rad) https://www.bio-rad.com/en-us/sku/1705047-immun-star-goat-anti-mouse-gam-hrp-conjugate?ID=1705047

IRDye® 800CW Goat anti-Mouse IgG Secondary Antibody (Cat#P/N: 926-32210, RRID AB_621842

Licor) https://www.licor.com/bio/reagents/irdye-800cw-goat-anti-mouse-igg-secondary-antibody

IRDye® 800CW Goat anti-Mouse IgG Secondary Antibody (Cat#P/N: 926-32210, RRID AB_621842,Licor) https://www.licor.com/bio/reagents/irdye-800cw-goat-anti-mouse-igg-secondary-antibody

Eukaryotic cell lines

Policy information about cell lines

Cell line source(s)	All RPE cells were derived from human iPSC cells (iPSC #1-10, Table 1) and Nemo-iPSCs. iPSCs were generated at NIH
Authentication	All cell lines were authenticated with immunostaining for pluripotency markers, three germ layer differentiation and karyotyping. Data is provided in Table S1.
Mycoplasma contamination	All cell lines were routinely tested to be negative for mycoplasma contamination
Commonly misidentified lines (See ICLAC register)	No commonly misidentified lines were used

Human research participants

Policy information about studies involving human research participants

Population characteristics	All participants were >65 years in age (because of AMD diagnosis), both genders were represented in recruited participants. Recruitment was based on their diagnosis of AMD. Healthy volunteers were age and gender matched but didnt have AMD or other ocular conditions.
Recruitment	Participants recruitment was based on AMD diagnosis. Healthy volunteers didn't have AMD or other ocular conditions. Patient recruitment was not biased in anyway. As per IRB rules, participant cells were anonymized for use in this study.
Ethics oversight	Clinical protocol and the consent forms were approved by Combined NeuroScience Institutional Review Board (CNS IRB) under the Office of Human Research Protection (OHRP), NIH as per 45 CFR 46 guidelines of U.S. Government approved the study

Note that full information on the approval of the study protocol must also be provided in the manuscript.
