# Supplementary material for: Epithelial phenotype restoring drugs suppress macular degeneration phenotypes in an iPSC model
Source: Nat Commun. 2021 Dec 15;12:7293. doi: 10.1038/s41467-021-27488-x (PMC8674335; doi:10.1038/s41467-021-27488-x)
Supplement: Supplementary file 1 — Supplementary Information [file 41467_2021_27488_MOESM1_ESM.pdf]

Supplementary Data – Sharma et al

Epithelial phenotype restoring drugs suppress macular degeneration phenotypes in an iPSC model

Ruchi Sharma[1], Aman George[2#], Malika Nimmagadda[1#], Davide Ortolan[1], Barbosa-Sabanero Karla[1], Zoya Qureshy[1], Devika Bose[1], Roba Dejene[1], Genqing Liang[1], Qin Wan[1], Justin Chang[1], Balendu Shekhar Jha[1], Omar Memon[1], Kiyoharu Joshua Miyagishima[1], Aaron Rising[1], Madhu Lal[3], Eric Hanson[4], Rebecca King[5], Mercedes Maria Campos[6], Marc Ferrer[5], Juan Amaral[1], David McGaughey[7], and Kapil Bharti[1,*]

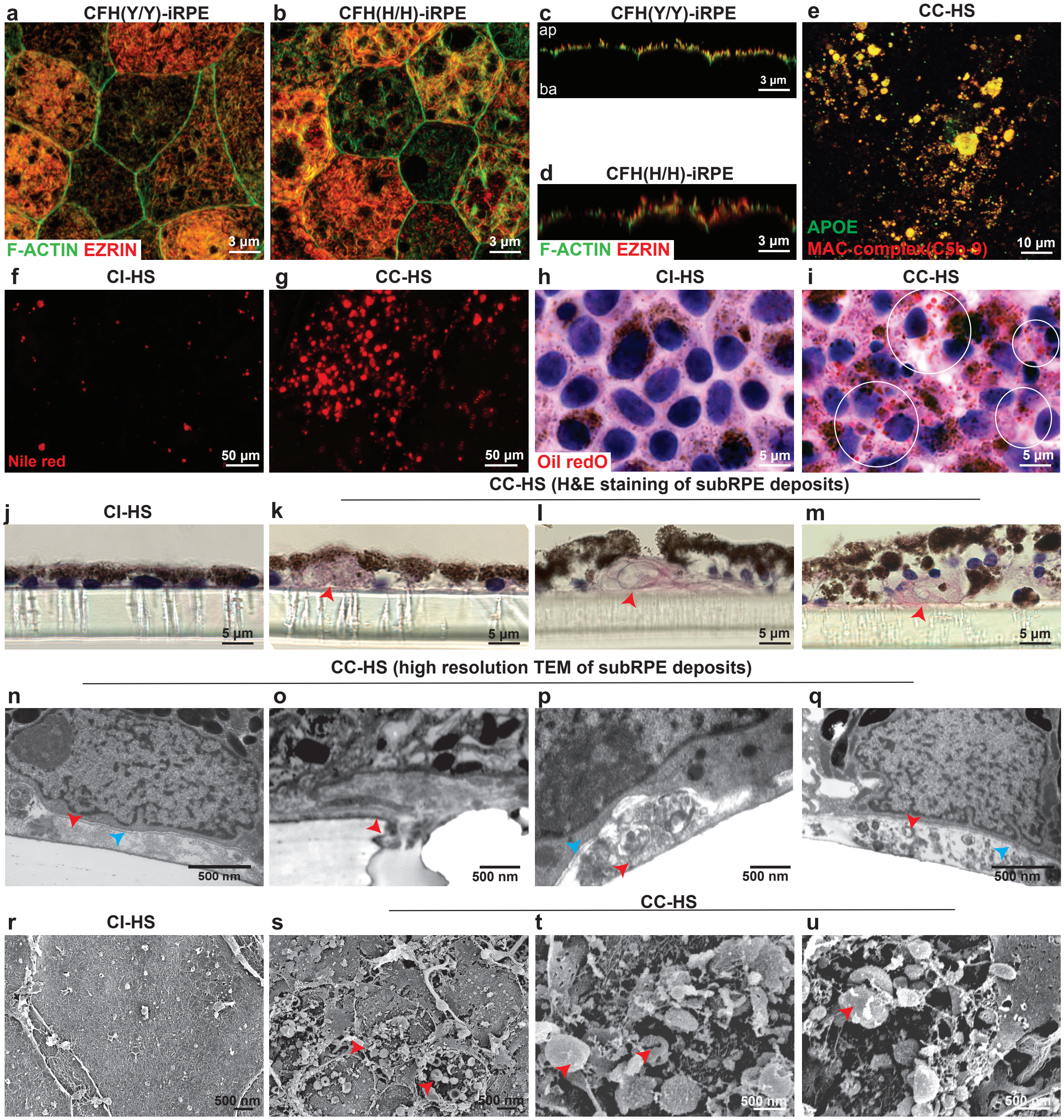

a
CFH(Y/Y)-iRPE
F-ACTIN EZRIN
3 µm
b
CFH(H/H)-iRPE
3 µm
c
CFH(Y/Y)-iRPE
ap
ba
3 µm
d
CFH(H/H)-iRPE
F-ACTIN EZRIN
3 µm
e
CC-HS
APOE
MAC complex(C5b-9)
10 µm
f
CI-HS
Nile red
50 µm
g
CC-HS
50 µm
h
CI-HS
Oil redO
5 µm
i
CC-HS
5 µm
CC-HS (H&E staining of subRPE deposits)
j
CI-HS
5 µm
k
5 µm
l
5 µm
m
5 µm
CC-HS (high resolution TEM of subRPE deposits)
n
500 nm
o
500 nm
p
500 nm
q
500 nm
CC-HS
r
CI-HS
500 nm
s
500 nm
t
500 nm
u
500 nm

Supplementary Fig. 1 Treatment of iRPE with complement competent human serum (CC-HS) leads to the formation of sub-RPE deposits in mature CFH(Y/Y)-iRPE and CFH(H/H)-iRPE. a-d Apical localization of EZRIN (red) in CFH (Y/Y)-iRPE (**a, c**) and CFH (H/H)-iRPE (**b, d**). F-ACTIN (green) marks cell borders and apical side of cells (N = 4 biologically independent replicates, iRPE 1, 2, 4, 7); ap - apical, ba - basal. **e** CC-HS treated transwell membrane co-stained for APOE (red) and C5b-9 (green). Yellow signal shows colocalization of the two signals (red + green). (N = 3 biologically independent replicates, iRPE 1, 4, 8). **f-i** Nile red (**f, g**) and Oil Red O (**h, i**—white circles) positive lipid droplets in CC-HS treated iRPE, compared to CI-HS treated iRPE (N = 8 biologically independent replicates, iRPE 1, 2, 3, 4, 5, 6, 7, 8 for Nile red; N = 6 biologically independent replicates, iRPE1, 4, 5, 6, 7, 8 for Oil Red O). **j-m** Cross-section images of CI-HS (**j**) or CC-HS (**k-m**) treated H&E stained iRPE show deposits specifically in CC-HS treated samples (red arrowhead, **k-m**) (N = 5 biologically independent replicates, iRPE 1, 4, 5, 6, 7). **n-q** High resolution TEM images of iRPE cross-sections show that deposits (red arrowheads) are clearly located under the RPE basal membrane (blue arrowheads), in between the RPE cell and the transwell membrane. In some cases, laminar shaped deposits (**n**) or deposits blocking the membrane pores (**o**) (N = 5 biologically independent replicates, iRPE 2, 4, 5, 8, 9). **r-u** Low (**r, s**) and high (**t, u**) resolution scanning electron micrographs of the sub-RPE region of iRPE following CI-HS (**r)** or CC-HS treatment (**s-u**; red arrowheads). (N = 3 biologically independent replicates, iRPE 1, 4, 8).

Supplementary Figure 2 Sharma et al

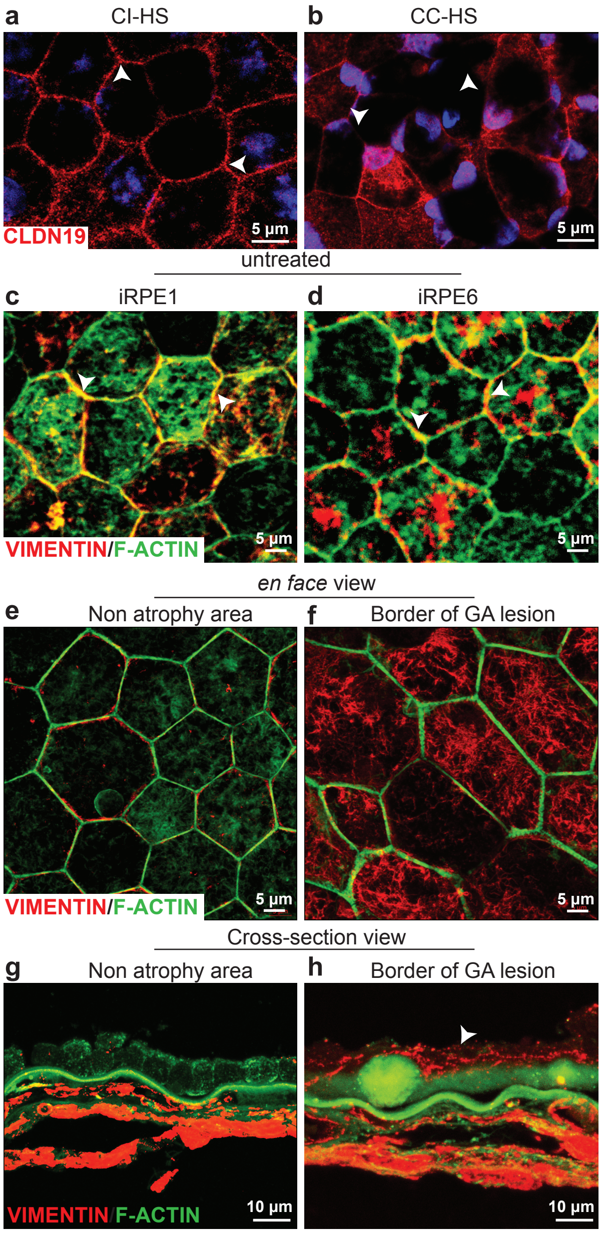

Supplementary Fig. 2 Treatment of iRPE with complement competent human serum (CC-HS) leads to the loss of iRPE epithelial phenotype in CFH(Y/Y)-iRPE and CFH(H/H)-iRPE. **a, b** Mis-localized expression of tight junction marker CLDN19 (red) in the cytoplasm (white arrowheads) of CC-HS treated iRPE, compared to CI-HS treated (N = 3 biologically independent replicates, iRPE 1, 5, 8). **c, d** Immunostaining for VIMENTIN (red) in untreated iRPE1 [CFH(Y/Y)] and iRPE6 [CFH/H/H)]. Arrowheads point to membrane staining (N = 6 biologically independent replicates, iRPE 1, 3, 5, 6, 7, 8). **e-h** *en-face* (**e, f**) and cross-section (**g, h**) views of immunostained VIMENTIN (red) in the non-atrophy area (**e, g**), of a cadaver AMD eye (98 years old female) and at the borders of geographic atrophy (GA) lesion in the same eye (**f, h**) (N = 3 independent immunostainings).

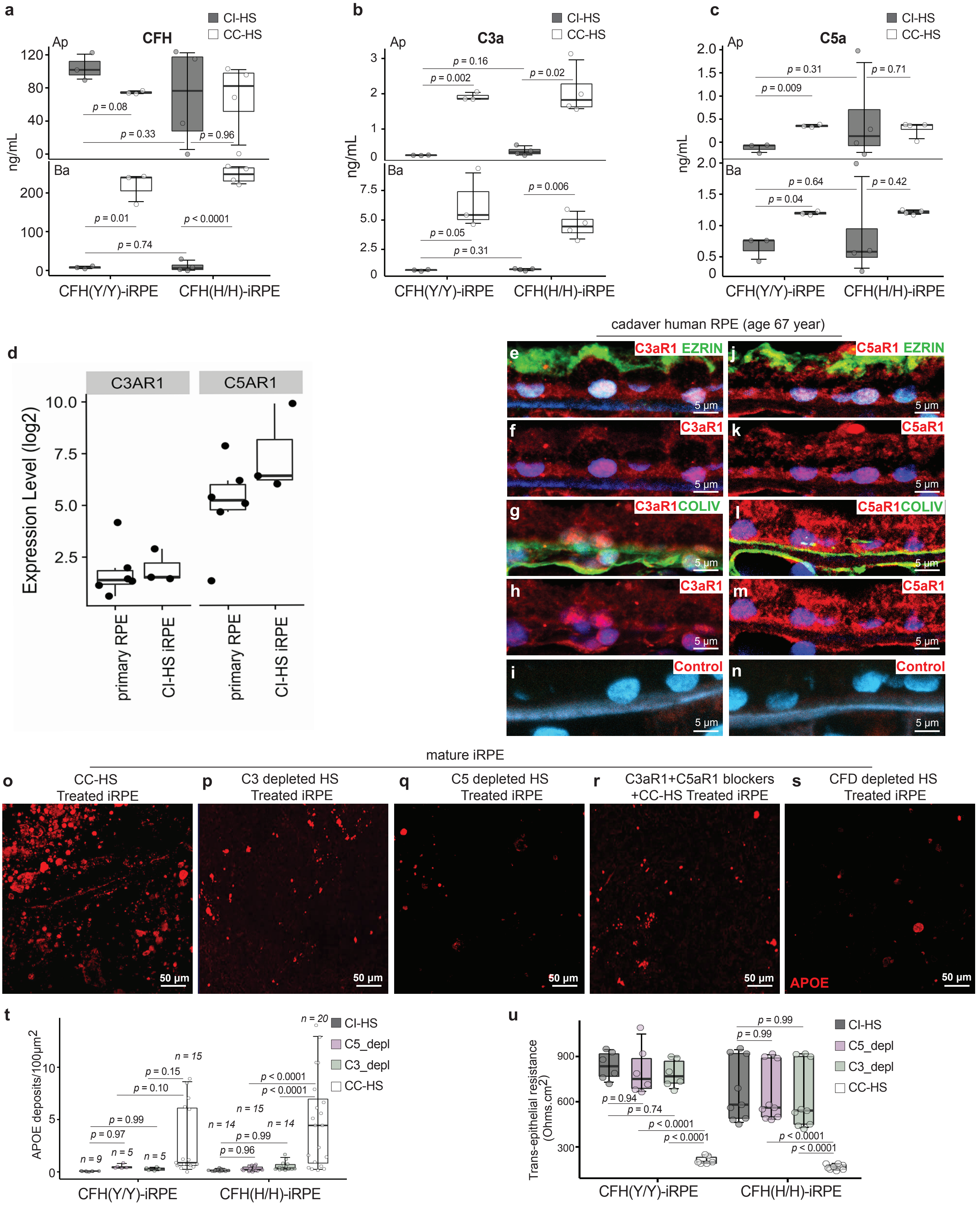
a
CFH
CI-HS
CC-HS
Ap
Ba
ng/mL
p = 0.08
p = 0.33
p = 0.96
p = 0.01
p < 0.0001
p = 0.74
CFH(Y/Y)-iRPE
CFH(H/H)-iRPE
b
C3a
p = 0.16
p = 0.002
p = 0.02
p = 0.05
p = 0.31
p = 0.006
c
C5a
p = 0.31
p = 0.009
p = 0.71
p = 0.64
p = 0.04
p = 0.42
d
C3AR1
C5AR1
Expression Level (log2)
primary RPE
CI-HS iRPE
cadaver human RPE (age 67 year)
e C3aR1 EZRIN
j C5aR1 EZRIN
f C3aR1
k C5aR1
g C3aR1 COLIV
l C5aR1 COLIV
h C3aR1
m C5aR1
i Control
n Control
5 µm
mature iRPE
o CC-HS Treated iRPE
p C3 depleted HS Treated iRPE
q C5 depleted HS Treated iRPE
r C3aR1+C5aR1 blockers +CC-HS Treated iRPE
s CFD depleted HS Treated iRPE
50 µm
APOE
t
APOE deposits/100µm2
CI-HS
C5_depl
C3_depl
CC-HS
n = 9
n = 5
n = 5
n = 15
n = 14
n = 15
n = 14
n = 20
p = 0.99
p = 0.97
p = 0.15
p = 0.10
p = 0.99
p = 0.96
p < 0.0001
p < 0.0001
u
Trans-epithelial resistance (Ohms.cm2)
p = 0.94
p = 0.74
p < 0.0001
p < 0.0001
p = 0.99
p = 0.99
p < 0.0001
p < 0.0001

Supplementary Fig. 3 Anaphylatoxins proteins mediate AMD cellular phenotypes in iRPE. a-c ELISA detects apically and basally secreted complement factor H (CFH) (**a**), C3a (**b**), and C5a (**c**) in CFH(Y/Y)-iRPE and CFH(H/H)-iRPE under CI-HS and CC-HS conditions [N = 7 biologically independent replicates iRPE 1, 2, 3, 4, 5, 6, 7; n = 3 technical replicates for CFH(Y/Y) and n = 4 for CFH(H/H)]. *P* values were determined by multiple pairwise comparisons using two-tailed t-test with 95% confidence interval and Bonferroni correction. The horizontal lines in the boxplots indicate the median, the boxes indicate the first and third quartiles, and the whiskers indicate 5th and 95th percentile. ap – apical, ba - basal. **d** mRNA expression levels for C3aR1 and C5aR1 in primary and iRPE cells. Note, ~30x higher expression of C5aR1 as compared to C3aR1 in iRPE cells. (N=3 biologically independent replicates, iRPE 1, 4, 8). The horizontal lines in the boxplots indicate the median, the boxes indicate the first and third quartiles, and the whiskers indicate 5th and 95th percentile. **e-n** In cadaver human eyes (67 year old donor), both C3aR1(red) and C5aR1(red) are expressed similarly on apical and basal sides of RPE cells, as confirmed by co-localization with EZRIN (green, apical marker) and with COLLAGEN IV (green, basal marker). There is also prominent intracellular expression of these receptors in RPE cells. Panels **f, h, k** and **m** are single channel images of panels **e, g, j,** and **l** respectively (N = 3 independent immunostainings). **o-s** APOE staining of iRPE membranes treated with human sera depleted of proteins C3, C5, co-treated with receptor blockers for C3aR1 and C5aR1, or serum depleted of protein CFD (upstream of C3 and C5), show reduced APOE expression under all conditions, compared to CC-HS treatment (N= 7 biologically independent replicates, iRPE 1, 2, 3, 4, 5, 6, 8). **t** APOE positive sub-RPE deposits in CFH(Y/Y) and CFH (H/H)-iRPE monolayers treated with CI-HS, C3 or C5 depleted sera, or CC-HS (N = 7 biologically independent replicates iRPE 1, 2, 3, 4, 5, 6, 7). *P* values were determined by pairwise comparisons using two-tailed t-test with 95% confidence interval and Bonferroni correction. **u** TER measurement across CFH(Y/Y) and CFH (H/H)-iRPE monolayers treated with CI-HS, C3 or C5 depleted sera, and CC-HS (N = 6 independent experiments for CFH(Y/Y) and N= 9 for CFH (H/H), iRPE 1, 2, 3, 4, 5, 6, 7). *P* values were determined by multiple pairwise comparisons using two-tailed t-test with 95% confidence interval and Bonferroni correction. The

horizontal lines in the boxplots indicate the median, the boxes indicate the first and third quartiles, and the whiskers indicate 5th and 95th percentile.

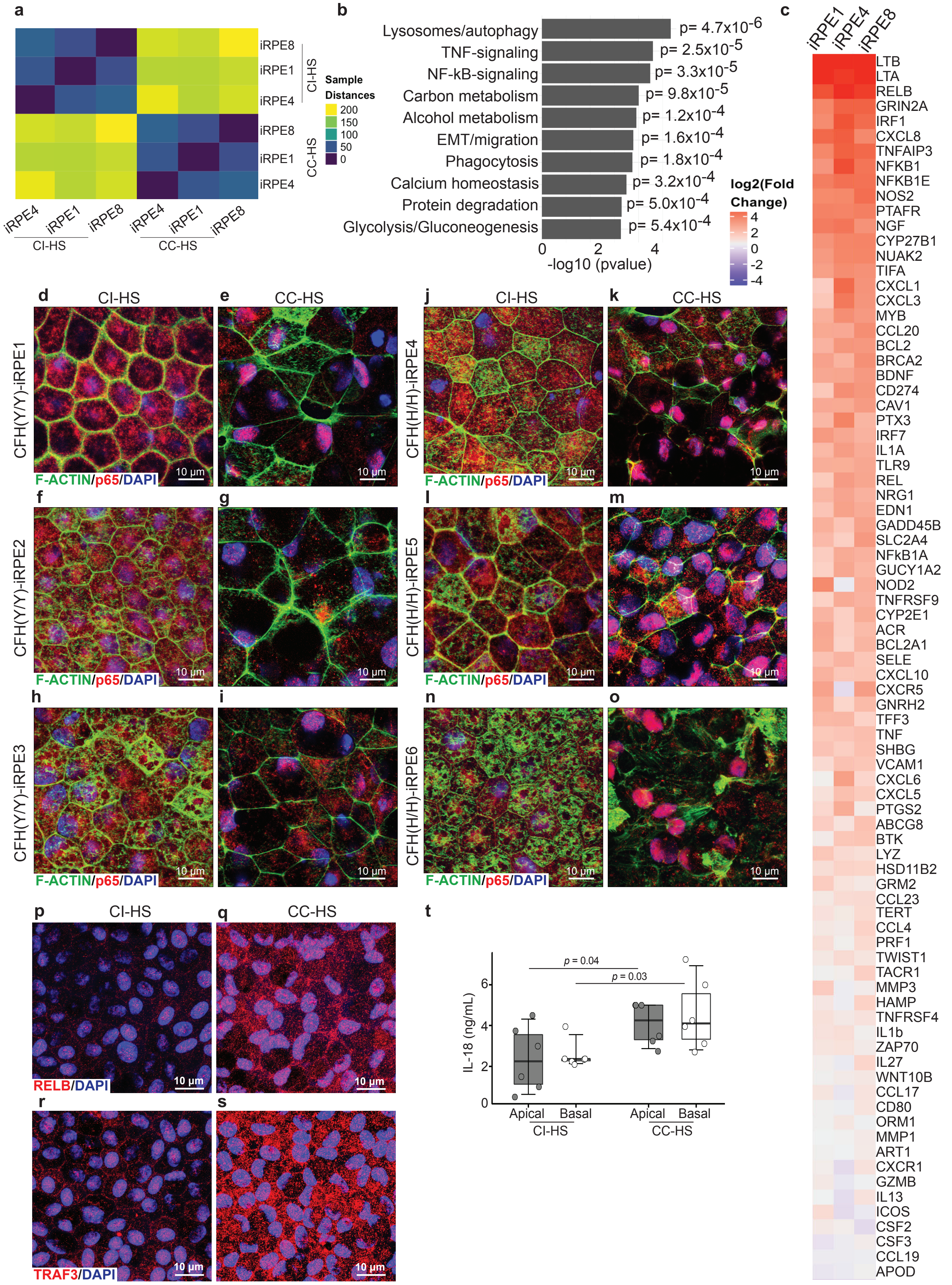
a
iRPE8
iRPE1
iRPE4
iRPE8
iRPE1
iRPE4
CI-HS
CC-HS
Sample Distances
200
150
100
50
0
iRPE4 iRPE1 iRPE8 iRPE4 iRPE1 iRPE8
CI-HS
CC-HS
b
Lysosomes/autophagy p= 4.7x10-6
TNF-signaling p= 2.5x10-5
NF-kB-signaling p= 3.3x10-5
Carbon metabolism p= 9.8x10-5
Alcohol metabolism p= 1.2x10-4
EMT/migration p= 1.6x10-4
Phagocytosis p= 1.8x10-4
Calcium homeostasis p= 3.2x10-4
Protein degradation p= 5.0x10-4
Glycolysis/Gluconeogenesis p= 5.4x10-4
0 2 4
-log10 (pvalue)
log2(Fold Change)
4
2
0
-2
-4
c
iRPE1 iRPE4 iRPE8
LTB
LTA
RELB
GRIN2A
IRF1
CXCL8
TNFAIP3
NFKB1
NFKB1E
NOS2
PTAFR
NGF
CYP27B1
NUAK2
TIFA
CXCL1
CXCL3
MYB
CCL20
BCL2
BRCA2
BDNF
CD274
CAV1
PTX3
IRF7
IL1A
TLR9
REL
NRG1
EDN1
GADD45B
SLC2A4
NFkB1A
GUCY1A2
NOD2
TNFRSF9
CYP2E1
ACR
BCL2A1
SELE
CXCL10
CXCR5
GNRH2
TFF3
TNF
SHBG
VCAM1
CXCL6
CXCL5
PTGS2
ABCG8
BTK
LYZ
HSD11B2
GRM2
CCL23
TERT
CCL4
PRF1
TWIST1
TACR1
MMP3
HAMP
TNFRSF4
IL1b
ZAP70
IL27
WNT10B
CCL17
CD80
ORM1
MMP1
ART1
CXCR1
GZMB
IL13
ICOS
CSF2
CSF3
CCL19
APOD
d CI-HS
e CC-HS
j CI-HS
k CC-HS
CFH(Y/Y)-iRPE1
CFH(H/H)-iRPE4
CFH(Y/Y)-iRPE2
CFH(H/H)-iRPE5
CFH(Y/Y)-iRPE3
CFH(H/H)-iRPE6
f g l m h i n o
F-ACTIN/p65/DAPI
10 µm
p CI-HS
q CC-HS
r s
RELB/DAPI
TRAF3/DAPI
t
p = 0.04
p = 0.03
IL-18 (ng/mL)
0 2 4 6
Apical Basal
CI-HS
Apical Basal
CC-HS

Supplementary Fig. 4 Increased NF-κB activation in CC-HS treated CFH(Y/Y)-iRPE and CFH(H/H)-iRPE. a Heatmap of RNAseq data from CFH(Y/Y), CFH (H/H) and CFH (Y/H) reveals clustering of samples according to CI-HS and CC-HS treatments (N = 3 biologically independent replicates, iRPE 1, 4, 8). Exact p-values are listed in the figures and are generated from a hypergeometric test from the enrichGO function using ClusterProfiler version 3.4.4. **b** Top ten pathways with statistically different gene expression pattern for CI-HS and CC-HS treated iRPE (N = 3 biologically independent replicates, iRPE1, 4, 8). **c** Heatmap of RNAseq data of up and down regulated NF-κB target genes in CC-HS treated iRPE normalized to CI-HS treated (N = 3 biologically independent replicates, iRPE 1, 4, 8). **d-o** Immunostaining reveals p65 nuclear localization in CC-HS treated CFH(Y/Y)-iRPE (N = 3 biologically independent replicates, iRPE 1, 2, 3; **d-i**) and CFH(H/H)-iRPE (N = 3 biologically independent replicates, iRPE 4, 5, 6; **j-o**; p65 – red; F-ACTIN – green; DAPI - blue). **p-s** Immunostaining for NF-κB target genes RELB (red) (**p, q**) and TRAF3 (red) (**r, s**) in CC-HS treated iRPE compared to CI-HS treated cells. (N=3 biologically independent replicates, iRPE1, 4, 8). **t** Quantification of ELISA results show a two-fold upregulation in apical and basal secretions of IL-18 an NF-κB downstream cytokine in CC-HS treated iRPE (N = 3 independent experiments and N=2 biological replicates, iRPE1, 9). *P* values were determined by pairwise comparisons using two-tailed t-test with 95% confidence interval and Bonferroni correction. The horizontal lines in the boxplots indicate the median, the boxes indicate the first and third quartiles, and the whiskers indicate 5th and 95th percentile.

Supplementary Figure 5 Sharma et al

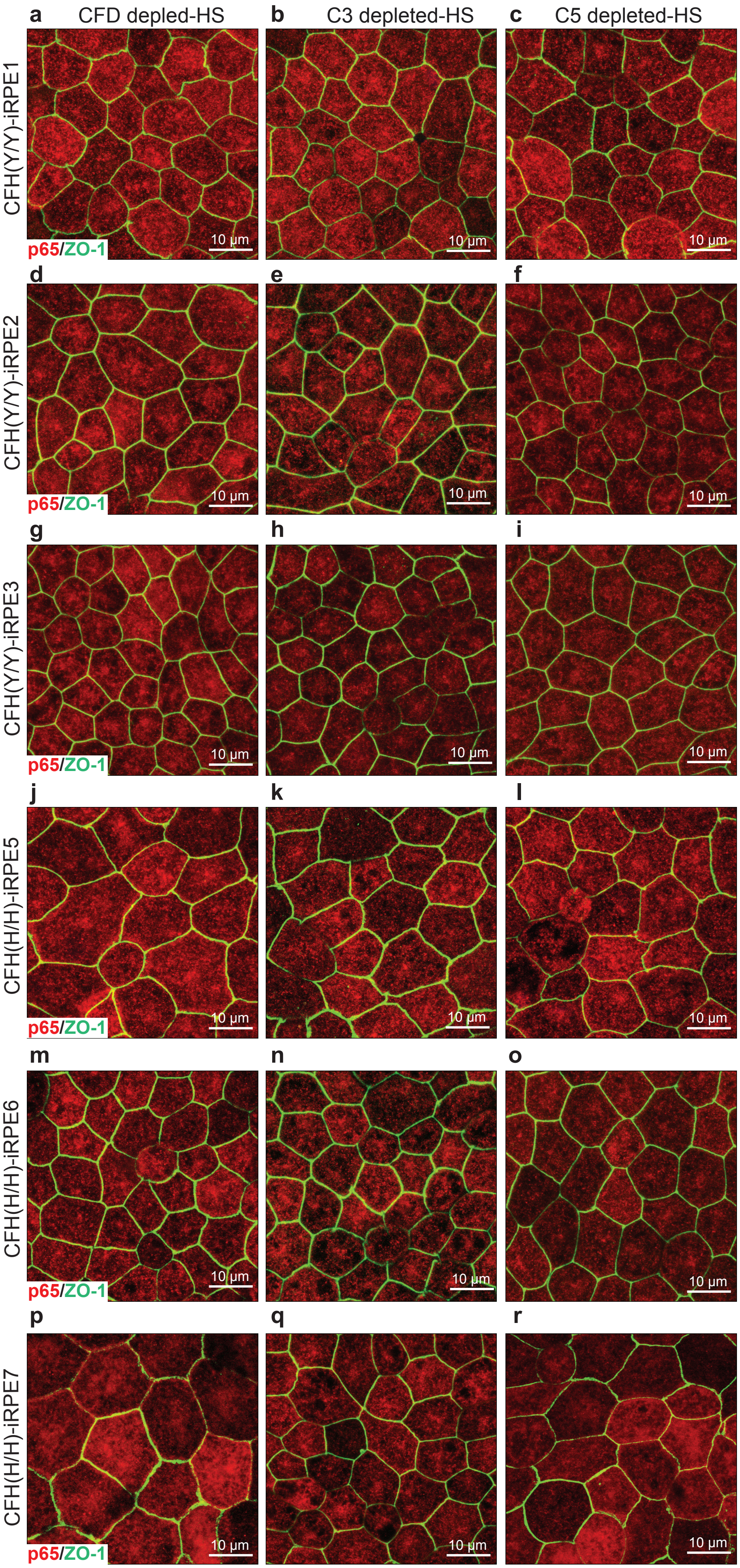

Supplementary Fig. 5 Anaphylatoxins drive NF-κB activation in CC-HS treated CFH(Y/Y)-iRPE and CFH(H/H)-iRPE. a-r iRPE cells treated with sera depleted in complement factor D (CFD), C3, or C5 show predominant cytoplasmic localization of p65 (red), ZO-1 (green). (N = 6 biologically independent replicates, iRPE 1, 2, 3, 5, 6, 7).

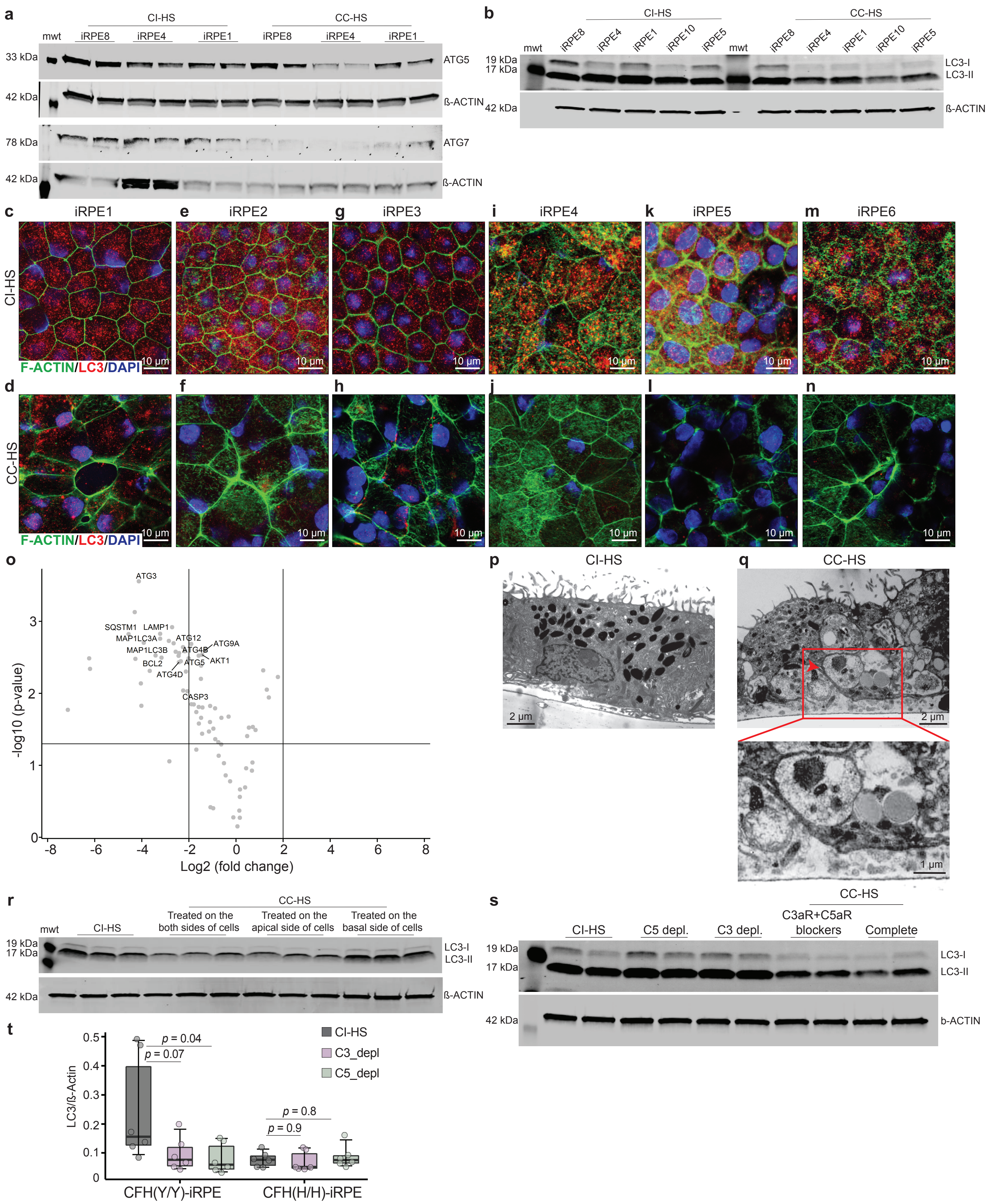
a
CI-HS
CC-HS
mwt
iRPE8
iRPE4
iRPE1
33 kDa
ATG5
42 kDa
ß-ACTIN
78 kDa
ATG7
b
iRPE10
iRPE5
19 kDa
17 kDa
LC3-I
LC3-II
c iRPE1
e iRPE2
g iRPE3
i iRPE4
k iRPE5
m iRPE6
d
f
h
j
l
n
F-ACTIN/LC3/DAPI
10 µm
o
ATG3
SQSTM1
LAMP1
MAP1LC3A
ATG12
MAP1LC3B
ATG4B
ATG9A
BCL2
ATG5
AKT1
ATG4D
CASP3
-log10 (p-value)
Log2 (fold change)
p CI-HS
q CC-HS
2 µm
1 µm
r
Treated on the both sides of cells
Treated on the apical side of cells
Treated on the basal side of cells
s
C5 depl.
C3 depl.
C3aR+C5aR blockers
Complete
b-ACTIN
t
LC3/ß-Actin
p = 0.04
p = 0.07
p = 0.8
p = 0.9
CI-HS
C3_depl
C5_depl
CFH(Y/Y)-iRPE
CFH(H/H)-iRPE

Supplementary Fig. 6 Anaphylatoxin signaling downregulates autophagy in CFH(Y/Y)-iRPE and CFH(H/H)-iRPE. a, b Western blots show reduced levels of autophagy proteins, ATG5 (**a**), ATG7 (**a**), and LC3-II (**b**), across CFH(Y/Y)-iRPE and CFH(H/H)-iRPE, upon CC-HS treatment. β-ACTIN was used for normalization. (N = 3 biologically independent replicates, iRPE 1, 4, 8 for **a**; N = 5 biologically independent replicates, iRPE 1, 4, 5, 8, 10 for **b**). **c-n** Immunostaining reveals reduced LC3 levels in CC-HS treated iRPE with (N =6 biologically independent replicates, iRPE 1, 2, 3, 4, 5, 6; LC3 – red; F-ACTIN – green; DAPI - blue). **o** 3-6-fold decreased expression of autophagy pathway genes in CC-HS treated iRPE, compared to CI-HS treated iRPE (N = 4 independent experiments, iRPE1, 8). Genes with more than 4x expression changes and $p<0.05$ are deemed significant. Exact p-values are plotted in the figure; statistics was performed using2 tailed t-test. **p, q** TEM shows accumulation of autophagosomes (arrowheads and higher magnification inset show intracellular location) in CC-HS treated (**q**) iRPE as compared to CI-HS treated (**p**) iRPE. (N=3 biologically independent replicates, iRPE1, 5, 8). **r** Western blots show LC3-II expression levels reduced only in iRPE samples across both low and high-risk for AMD treated with CC-HS on the apical side, or both sides, but not when treated only on the basal side. β-ACTIN was used for normalization. (N=3 biologically independent replicates, iRPE 1, 5, 8). **s** Western blots showed similar LC3-II expression levels across CI-HS treated iRPE, iRPE treated with C5 or C3 depleted human serum, or iRPE co-treated with CC-HS and C5aR1+C3aR1 receptor blockers. β-ACTIN was used for normalization. (N = 5 independent experiments, iRPE1,4,5, 8,10). **t** Western blot quantification for LC3-II levels in iRPE treated with CI-HS, and sera depleted in C5 or C3 (N = 6 biologically independent replicates iRPE 1, 2, 3, 4, 5, 6). *P* values were determined by pairwise comparisons using two-tailed t-test with 95% confidence interval and Bonferroni correction. The horizontal lines in the boxplots indicate the median, the boxes indicate the first and third quartiles, and the whiskers indicate 5th and 95th percentile.

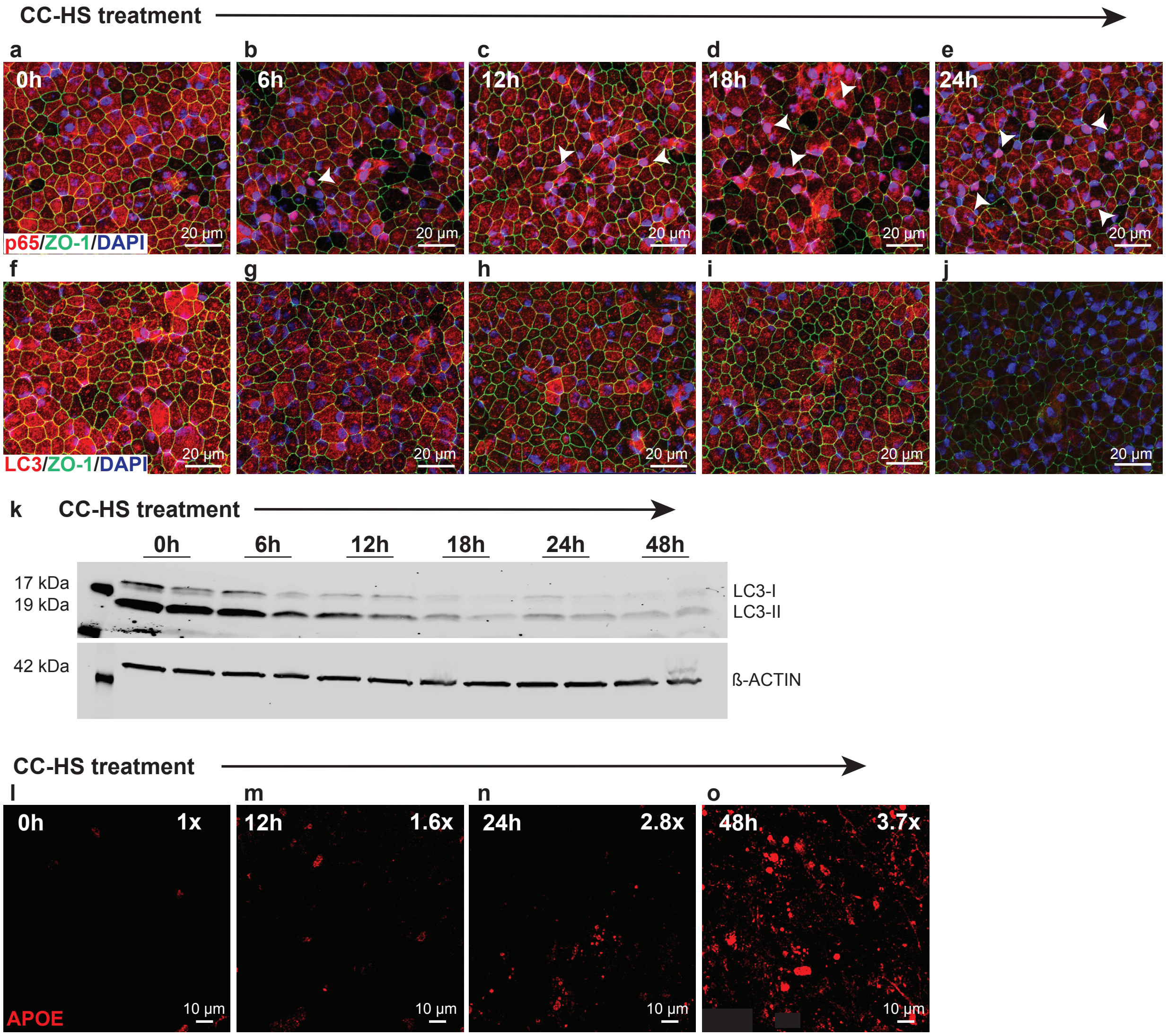
CC-HS treatment
a
b
c
d
e
0h
6h
12h
18h
24h
p65/ZO-1/DAPI
20 µm
f
g
h
i
j
LC3/ZO-1/DAPI
k
CC-HS treatment
0h
6h
12h
18h
24h
48h
17 kDa
19 kDa
42 kDa
LC3-I
LC3-II
ß-ACTIN
CC-HS treatment
l
m
n
o
0h
1x
12h
1.6x
24h
2.8x
48h
3.7x
APOE
10 µm

Supplementary Fig. 7 Time course of nuclear p65 translocation, downregulation of LC3-II levels, and APOE deposit formation in CC-HS treated iRPE. a-e Immunostaining shows a gradual increase in the nuclear localization of p65 (red) in iRPE following CC-HS treatment. Arrowheads point to nuclear p65 in a few cells 6 hours post CC-HS treatment. This number gradually increases and reaches maximum at around 24 hours. (N = 4 independent experiments, iRPE 1, 8). **f-k** Immunostaining (**f-j**) and Western blot (**k**) show a progressive decrease in LC3-II expression levels in iRPE following CC-HS treatment. Earliest decrease can be detected by 6 hours post CC-HS treatment. (N = 4 independent experiments, iRPE 1, 8 for **f-j**; N=3, biologically independent replicates, iRPE 1,6, 8 for **k**). **l-o** Immunostaining shows increasing APOE positive sub-RPE deposits in iRPE treated with increasing duration of CC-HS treatment. (N = 3 independent experiments, iRPE 8). 1.6x fold increase is seen by 12h, 2.8x by 24h, and 3.7x by 48h.

Supplementary Figure 8 Sharma et al

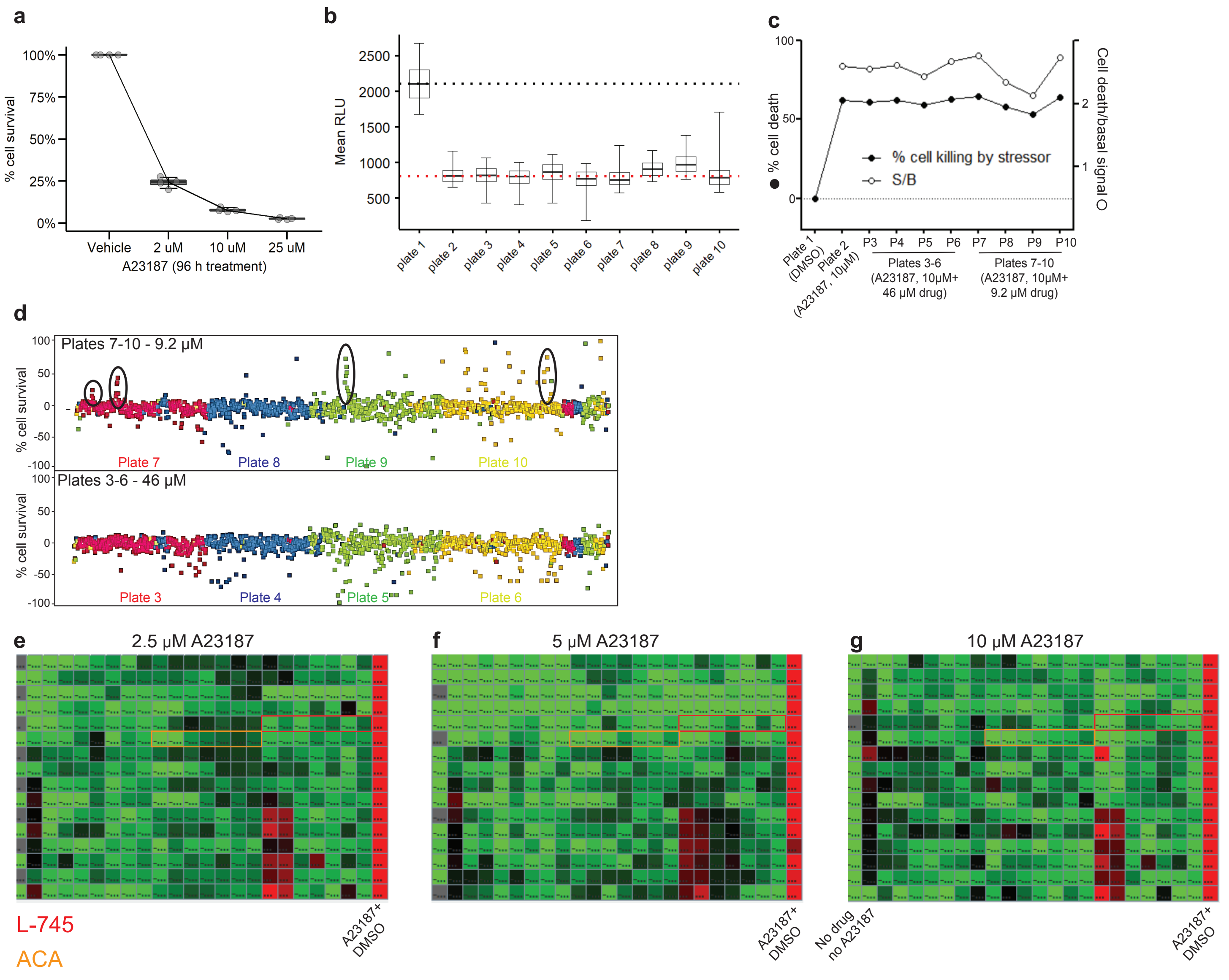

Supplementary Fig. 8 High throughput screen with iRPE cells. a Viability curve of iRPE cells treated with three concentrations of A23187 (2.5 µM, 10 µM, 25 µM). After 96 h, all three concentrations are cytotoxic to iRPE cells. (N = 4 independent experiments, iRPE4). **b** Mean relative light intensity units (RLU) shows similar mean signal across all plates treated with A23187 and vehicle, or A23187 and drugs at two different concentrations (9.2 µM and 46 µM), suggesting screen reproducibility across nine plates (n = 320 individual wells per plate, 9 independent plates, iRPE4). The horizontal lines in the boxplots indicate the median, the boxes indicate the first and third quartiles, and the whiskers indicate 5th and 95th percentile. The screen reproducibility is defined on strictly standardized mean difference (SSMD) compared to negative plate (Plate1) and positive plate(plate2). **c** Percent cell death caused by A23187 is similar across all plates containing vehicle or 46 µM, but slightly lower in plates treated with 9.2 µM drug, suggesting increased cell survival in those plates. **d** Dot plot shows results from two different sets of 384-well plates from the primary drug screen. In plates 3-6, iRPE were treated with 10 µM A23187 and 46 µM of each of the 1280 Library of Pharmaceutically Active Drugs (LOPAC) drugs, whereas in plates 7-10, iRPE were treated with 10 µM A23187 and 9.2 µM of each LOPAC drugs. At 9.2 µM concentration, 45 drugs improved iRPE cell survival to varying degrees. **e-g** A follow up screen confirmed two drugs (L745 and ACA) have reproducible cytoprotective effects across both CFH(Y/Y)-iRPE and CFH(H/H)-iRPE cells (N = 3 independent experiments, iRPE1 and 4). Heatmaps of results from the secondary screen using three different A23187 concentrations [2.5 µM – (**e**), 5 µM – (**f**), 10 µM – (**g**)] and seven different concentrations of drugs ranging from (0.78 µM - 50 µM). Responses of the two select drugs are highlighted (N = 3 independent experiments, iRPE1 and 4).

Supplementary Figure 9 Sharma et al

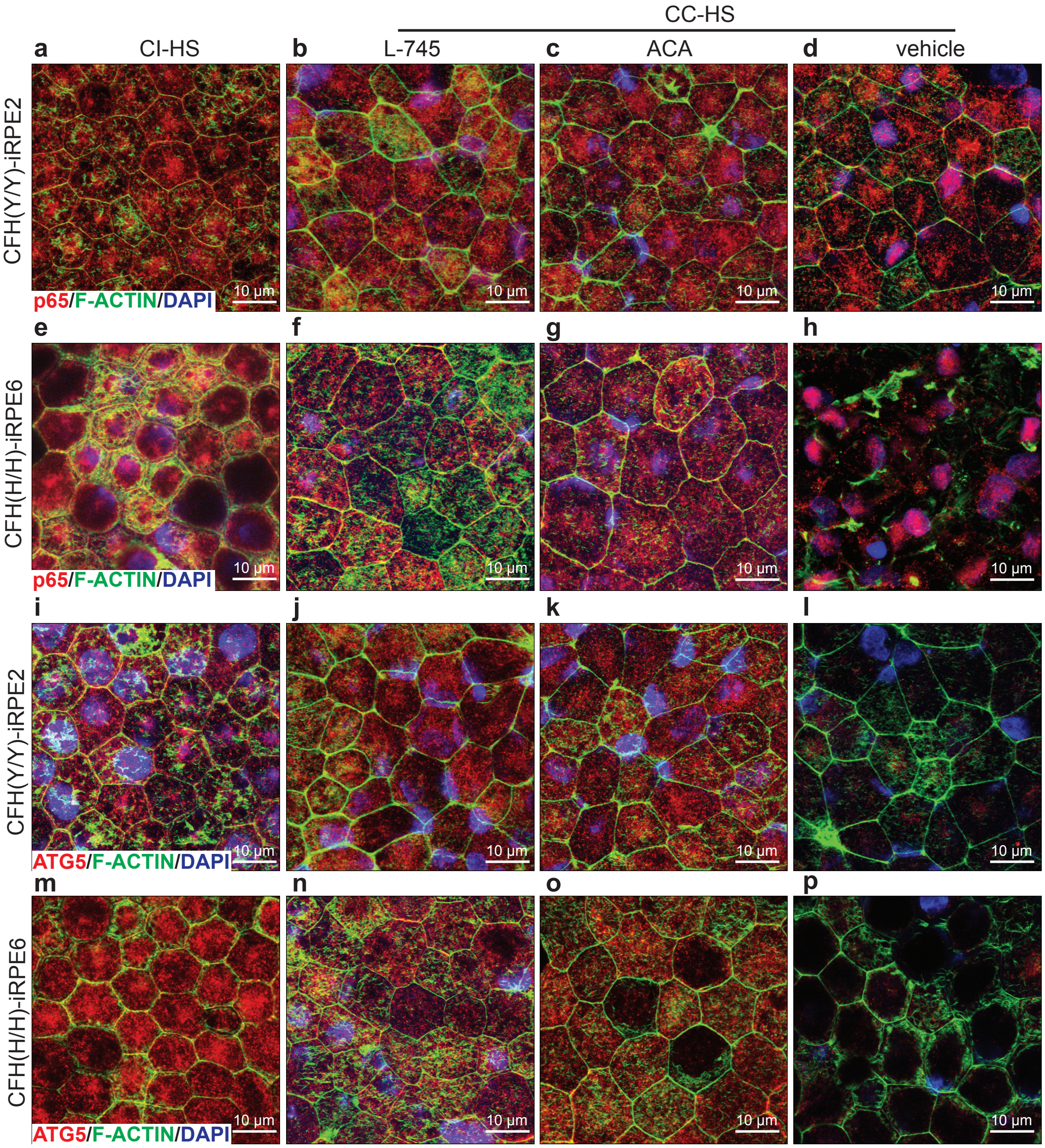

Supplementary Fig. 9 Restoration of NF-κB and autophagy pathways activity in CC-HS treated CFH(Y/Y)-iRPE and CFH(H/H)-iRPE. **a-h** L745 and ACA suppress nuclear translocation of p65 (red) induced by CC-HS treatment on CFH(Y/Y)-iRPE and CFH(H/H)-iRPE; F-ACTIN (green), DAPI (blue). (N = 4 biologically independent replicates, iRPE 1, 2, 5, 6). **i-p** L745 and ACA increase ATG5 (red) expression in CFH(Y/Y)-iRPE and CFH(H/H)-iRPE cells treated with CC-HS. F-ACTIN (green), DAPI (blue). (N = 4 biologically independent replicates, iRPE 1, 2, 5, 6).

Supplementary Figure 10 Sharma et al

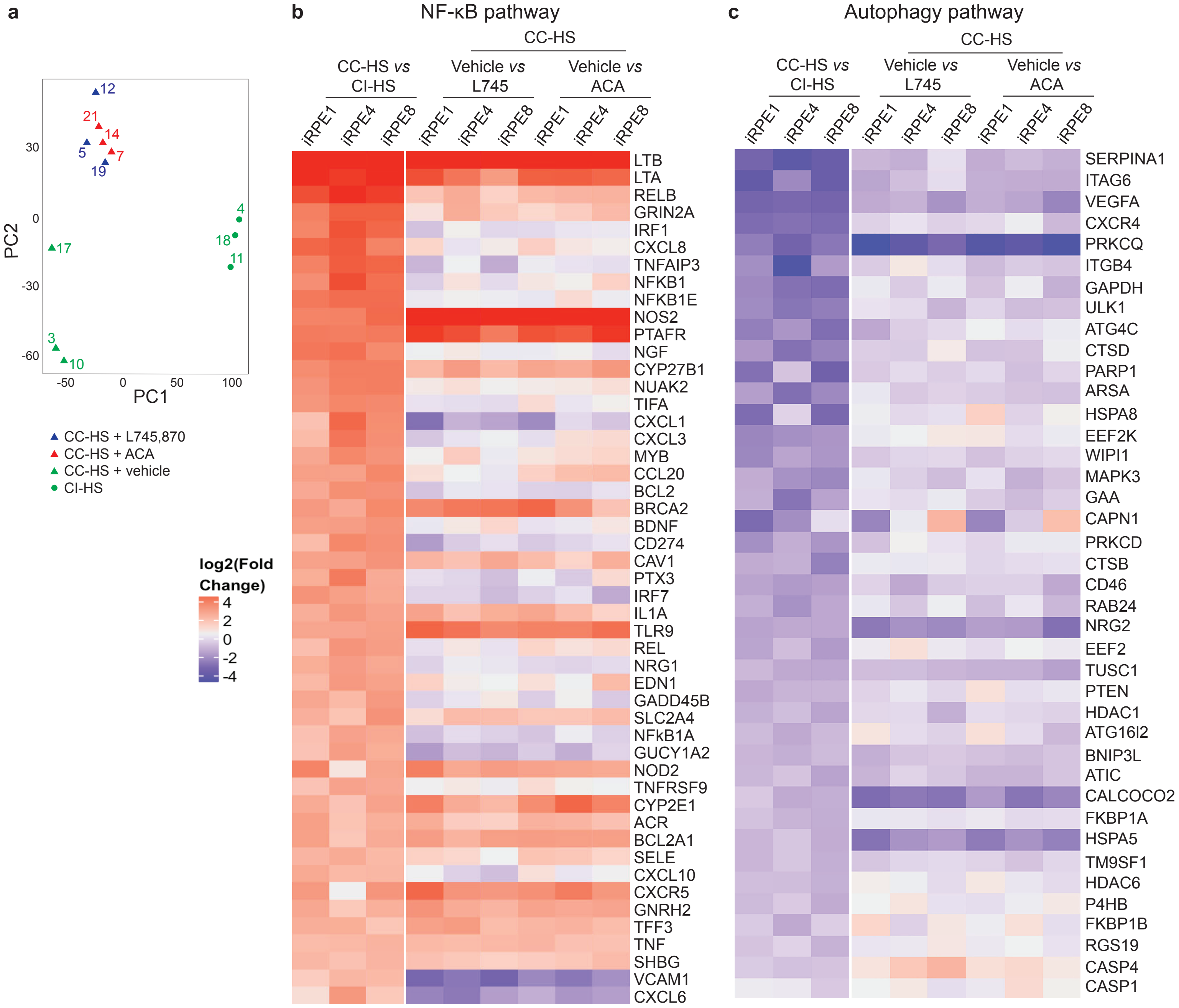

Supplementary Fig. 10 RNA seq analysis of drug treated CFH(Y/Y)-iRPE, CFH(Y/H)-iRPE and CFH(H/H)-iRPE cells. a PCA plot shows separate clustering for iRPE co-treated with drugs and CC-HS, only CC-HS, and only CI-HS. (N = 3 biologically independent replicates, iRPE 1, 4, 8). **b, c** Heatmaps of RNA seq data for iRPE derived from CFH(Y/Y)-iRPE, CFH(Y/H)-iRPE and CFH(H/H)-iRPE AMD samples and three treatment groups (CI-HS vs CC-HS, CC-HS+vehicle vs CC-HS+L745 or ACA) shows a reversal of the upregulation of NF-κB pathway genes and a reversal in downregulation of autophagy genes in samples co-treated with drugs and CC-HS as compared to samples treated with CC-HS. (N = 3 biologically independent replicates, iRPE 1, 4, 8).

Supplementary Figure 11 Sharma et al

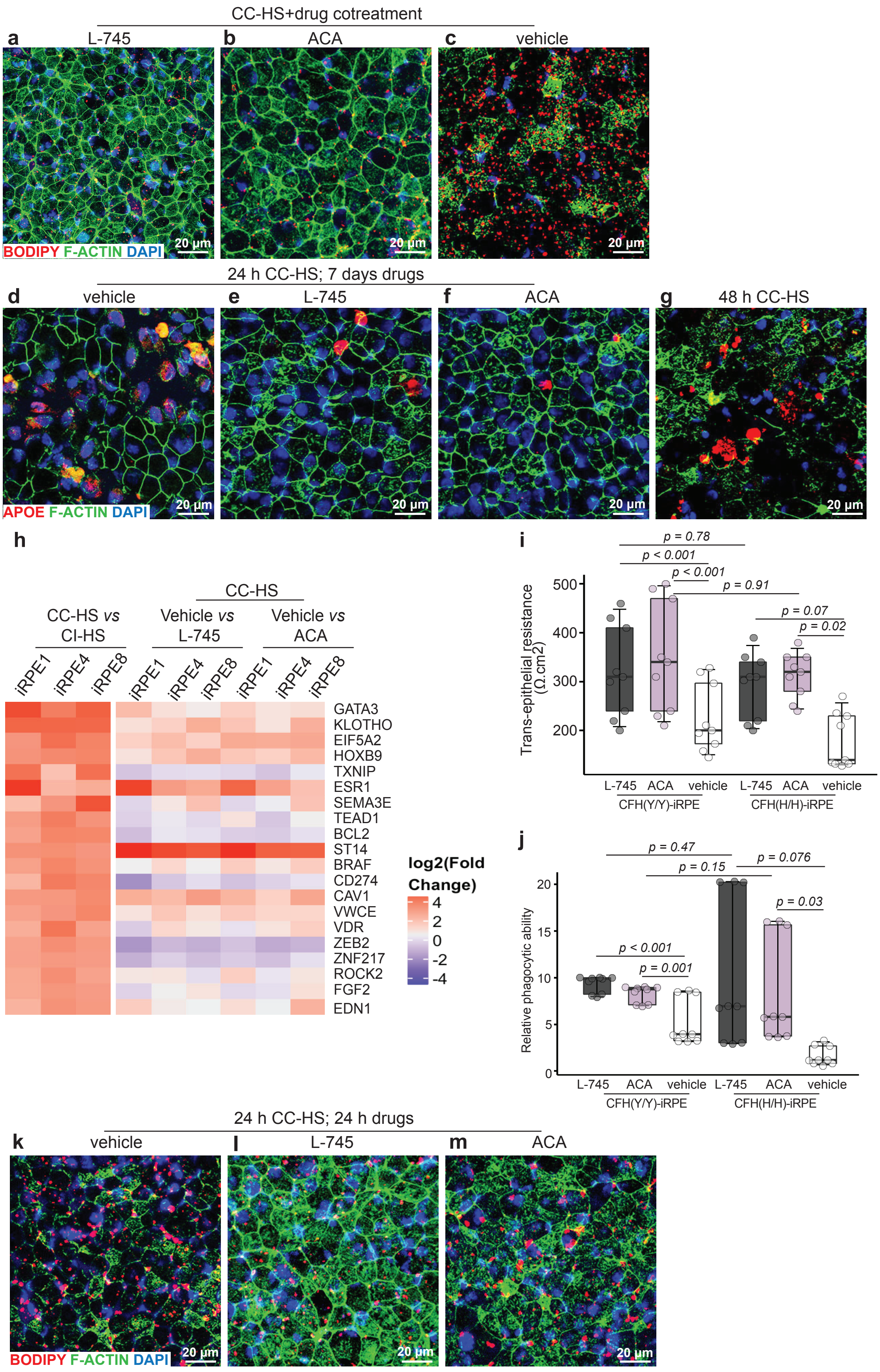

Supplementary Fig. 11 Drug treatment reduces sub-RPE lipid and APOE deposits and restores epithelial phenotype in CFH(Y/Y)-iRPE and CFH(H/H)-iRPE. **a-c** BODIPY (red) and F-ACTIN (green) staining in CFH(Y/Y), CFH(Y/H), and CFH(H/H)-iRPE co-treated with CC-HS plus L745 or aminocaproic acid (ACA), compared with CC-HS plus vehicle co-treated cells (N = 6 biologically independent replicates, iRPE 1, 4, 5, 6, 7, 8). **d-g** APOE (red) and F-ACTIN (green) staining in CFH(Y/Y), CFH(Y/H), CFH(H/H) iRPE treated with vehicle, L745 or ACA for seven days after a 24-hour CC-HS treatment, compared with a 48-hour CC-HS treatment (N= 6 biologically independent replicates, iRPE 1, 4, 5, 6, 7, 8). **h** Heatmap of RNA seq data for iRPE derived from three donors (N = 3 biologically independent replicates, iRPE 1, 4, 8) and three treatment groups (CI-HS vs CC-HS, CC-HS + vehicle vs CC-HS+L745 or ACA) shows that both drugs are effective in downregulating de-differentiation genes that are upregulated by CC-HS treatment of iRPE cells. **i, j** Co-treatment of CC-HS with drugs L745 or ACA rescued CC-HS induced TER drop (**i**) and improved phagocytic ability (**j**) in both CFH(Y/Y) and CFH(H/H)-iRPE (N = 6 biologically independent replicates iRPE 1, 2, 3, 5, 6, 7 and n = 9 technical replicates). *P* values were determined by multiple pairwise comparisons using two-tailed t-test with 95% confidence interval and Bonferroni correction. The horizontal lines in the boxplots indicate the median, the boxes indicate the first and third quartiles, and the whiskers indicate 5th and 95th percentile. **k-m** BODIPY (red) and F-ACTIN (green) staining in iRPE treated with vehicle, L745 or ACA after 24-hours of CC-HS treated (N = 6 biologically independent replicates, iRPE 1, 4, 5, 6, 7, 8).

Supplementary Figure 12 Sharma et al

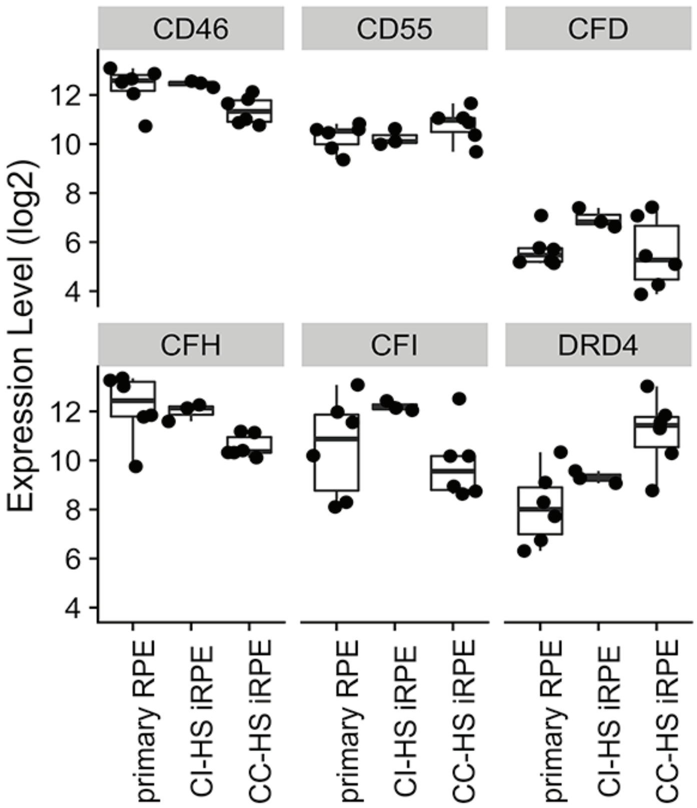

Supplementary Fig. 12 mRNA expression levels for complement regulators CD46, CD55, CFD, CFH, CFI, and DRD4 in CI-HS *vs* CC-HS treated cells reveal no significant change in gene expression in CC-HS treated cells derived from CFH(Y/Y) and CFH(H/H)-iRPE (N = 3 biologically independent replicates, iRPE 1, 4, 8). The horizontal lines in the boxplots indicate the median, the boxes indicate the first and third quartiles, and the whiskers indicate 5th and 95th percentile.

Supplementary References

1. Fritsche, L.G., Igl, W., Bailey, J.N., Grassmann, F., Sengupta, S., Bragg-Gresham, J.L., Burdon, K.P., Hebbring, S.J., Wen, C., Gorski, M., Kim, I.K., Cho, D., Zack, D., et al. (2016). A large genome-wide association study of age-related macular degeneration highlights contributions of rare and common variants. Nat Genet. *21*, 3227-36.

2. Maruotti, J., Sripathi, S.R., Bharti, K., Fuller, J., Wahlin, K.J., Ranganathan, V., Sluch, V.M., Berlinicke, C.A., Davis, J., Kim, C.*, et al.* (2015). Small-molecule-directed, efficient generation of retinal pigment epithelium from human pluripotent stem cells. Proc Natl Acad Sci U S A *112*, 10950-10955.

3. Sharma, R., Khristov, V., Rising, A., Jha, B.S., Dejene, R., Hotaling, N., Li, Y., Stoddard, J., Stankewicz, C., Wan, Q.*, et al.* (2019). Clinical-grade stem cell-derived retinal pigment epithelium patch rescues retinal degeneration in rodents and pigs. Sci Transl Med *11*.

4. Ounkomol, C., Seshamani, S., Maleckar, M.M., Collman, F., Johnson, G.R.(2018).Label-free prediction of three-dimensional fluorescence images from transmitted-light microscopy. Nat Methods *11*, 917-920.

5. Zilberman-Rudenko, J., Shawver, L.M., Wessel, A.W., Luo, Y., Pelletier, M., Tsai, W.L., Lee, Y., Vonortas, S., Cheng, L., Ashwell, J.D., *et al*. (2016). Recruitment of A20 by the C-terminal domain of NEMO suppresses NF-κB activation and autoinflammatory disease. Proc Natl Acad Sci U S A. *113*, 1612-7

Supplementary Table1. Summary of iPSC lines used in the study. All the iPSC lines were characterized for the expression of pluripotency by markers SSEA4, OCT4, and TRA1-81, and the expression of markers for all three germ layers using an *in vitro* three germ layer differentiation assay. iPSC derived RPE (iRPE) used in the study are numbered according to numbering of iPSC lines used in this Table

**iPSC lines**	**SSEA4 expression**	**OCT4 expression**	**TRA1-81 expression**	**Endoderm Gene Expression**	**Mesoderm Gene Expression**	**Ectoderm Gene Expression**
**iPSC with CFH(Y/Y) allele (Fritsche et al., 2016)**						
iPSC1	POSITIVE	POSITIVE	POSITIVE	POSITIVE	POSITIVE	POSITIVE
iPSC2	Sharma et al., 2019					
iPSC3	Sharma et al., 2019					
**iPSC with CFH(H/H) allele (Fritsche et al., 2016)**						
iPSC4	Maruotti et al., 2015					
iPSC5	Sharma et al., 2019					
iPSC6	Ounkomol et al., 2018					
iPSC7	Miyagishima Nature Commu. Biol (under revision)					
**iPSC with CFH(Y/H) allele (Fritsche et al., 2016)**						
iPSC8	POSITIVE	POSITIVE	POSITIVE	POSITIVE	POSITIVE	POSITIVE
iPSC9	POSITIVE	POSITIVE	POSITIVE	POSITIVE	POSITIVE	POSITIVE
iPSC10	POSITIVE	POSITIVE	POSITIVE	POSITIVE	POSITIVE	POSITIVE
**Nemo-iRPE**	**Zilberman-Rudenko et al., 2016**					
